# Habitat-Associated Variation in Dietary Composition, Gut Microbiota, and Muscle Nutritional Characteristics of *Hemibagrus guttatus* in a Dam-Regulated River

**DOI:** 10.3390/ani16182818

**Published:** 2026-09-08

**Authors:** Jiajun Zhang, Fangyuan Xiong, Mengyao Li, Shijie Wang, Xiaoya Wang, Li Wang, Yuansheng Zhu, Miao Li, Zechen E, Xuemei Li, Wanjie Cai, Hongfei Li

**Affiliations:** 1 Scientific Institute of Pearl River Water Resources Protection, Guangzhou 510611, China; 2 Hongshui River Rare Fish Conservation Center, Guigang 537200, China; 3 Engineering Research Center of Hongshui River Rare Fish Conservation, Guigang 537200, China; 4 Pingyang Institute of Science and Technology Innovation, No. 8 Binjiang Avenue, Aojiang Town, Pingyang, Wenzhou 325400, China; 5 National Engineering Research Center of Marine Facilities Aquaculture, Marine Science and Technology College, Zhejiang Ocean University, Zhoushan 316004, China

**Keywords:** *Hemibagrus guttatus*, gut microbiota, dietary metabarcoding, muscle nutritional composition, aquaculture nutrition, dam-regulated river

## Abstract

Hydropower regulation can alter riverine habitats and potentially influence fish diet, gut microbiota, and nutritional characteristics. In this study, upstream and downstream populations of *Hemibagrus guttatus* (*H. guttatus*) were investigated in a dam-regulated reach of the Hongshui River associated with the Datengxia Water Conservancy Project using 12S rRNA and COI metabarcoding, 16S rRNA gene sequencing, and muscle nutritional analyses. Fish from the two habitats exhibited distinct dietary compositions, accompanied by significant differences in gut microbial diversity, community structure, and predicted microbial functions. Upstream fish harbored higher microbial diversity and greater abundances of Firmicutes-associated taxa, including several putatively beneficial bacteria, whereas downstream fish showed enrichment of Proteobacteria-associated taxa and functions related to environmental adaptation. Muscle composition also differed between habitats, with upstream fish containing higher moisture and crude lipid contents and downstream fish exhibiting higher crude protein content. These habitat-associated differences suggest that natural dietary resources and intestinal microbiota are closely linked to host nutritional characteristics. These findings highlight the importance of considering upstream–downstream habitat origin when evaluating the trophic, microbial, and nutritional characteristics of *H. guttatus* and provide a biological reference for the management and conservation of fish populations in dam-regulated rivers.

## Simple Summary

Hydropower dams can alter river habitats and may be associated with changes in the feeding ecology and physiological characteristics of aquatic animals. This study compared wild spotted longbarbel catfish (*Hemibagrus guttatus*) collected from upstream and downstream areas of the Datengxia Water Conservancy Project in the Hongshui River, China. We investigated their natural diet, gut bacterial communities, and muscle nutritional composition to determine whether fish from the two areas showed distinct biological characteristics. The results revealed clear upstream–downstream differences in prey use, gut bacterial composition, and muscle nutritional traits. Upstream fish generally showed greater gut bacterial diversity and higher muscle fat content, whereas downstream fish had different dietary characteristics and higher muscle protein content. These findings indicate that fish from upstream and downstream habitats represent biologically distinct groups within the same regulated river system. The results provide useful baseline information for understanding wild populations of this protected species and may help guide the selection of fish for conservation breeding, stock enhancement, and the future development of nutritional management strategies for aquaculture.

## 1. Introduction

River damming associated with hydropower development has become one of the major anthropogenic drivers altering freshwater ecosystems worldwide [1,2]. The construction and operation of large dams can substantially modify natural hydrological regimes by changing flow patterns, water residence time, sediment transport, thermal conditions, and longitudinal river connectivity [3,4,5]. These alterations frequently generate ecological differences between upstream impounded areas and downstream riverine habitats, leading to changes in biological communities and ecosystem processes [6]. Following reservoir impoundment, upstream reaches often shift from lotic to relatively slow-flowing environments, whereas downstream sections may experience altered discharge regimes and hydrodynamic fluctuations caused by artificial flow regulation. Such environmental changes can influence habitat characteristics, resource availability, and trophic interactions within aquatic ecosystems [6,7].

Hydropower development has been shown to affect fish assemblages, population dynamics, and trophic structure in freshwater ecosystems [8]. Habitat fragmentation resulting from dam construction may restrict fish movement and alter the spatial distribution of prey resources, potentially leading to divergence in feeding strategies among populations inhabiting different river sections [9]. Moreover, environmental heterogeneity generated by river regulation can influence the abundance and composition of benthic organisms, planktonic resources, and detrital organic matter, thereby modifying energy transfer pathways within aquatic food webs [10]. Because fish occupy relatively high trophic positions in freshwater ecosystems, variation in dietary composition may provide valuable information regarding habitat conditions and resource utilization patterns in dam-regulated rivers.

Traditional studies of fish feeding ecology have largely relied on morphological identification of stomach contents. However, prey tissues are often highly degraded during digestion, making accurate taxonomic identification difficult and potentially resulting in an underestimation of dietary diversity. In recent years, DNA metabarcoding has emerged as a powerful approach for reconstructing dietary composition and trophic interactions in aquatic organisms [11]. By combining universal genetic markers with high-throughput sequencing technologies, metabarcoding enables the detection of prey taxa with high sensitivity and taxonomic resolution. In particular, mitochondrial markers such as 12S rRNA and cytochrome oxidase I (COI) have been widely applied in dietary studies of aquatic consumers and have substantially improved our understanding of trophic ecology and resource utilization in natural ecosystems [11]. Beyond dietary composition, intestinal microbial communities play important roles in digestion, nutrient assimilation, energy metabolism, immune regulation, and maintenance of host health [12,13]. Increasing evidence indicates that gut microbiota composition is strongly influenced by dietary resources, environmental conditions, and habitat characteristics [14]. Changes in food availability and feeding strategies may be accompanied by shifts in intestinal microbial communities, with possible consequences for nutrient utilization and host condition. Information obtained from natural diets and resident gut microbiota may also be useful for aquaculture, because it can help identify nutritionally relevant prey resources, indigenous bacterial taxa with probiotic potential, and population-specific nutritional baselines. However, few studies have jointly examined diet, gut microbiota, and muscle nutritional traits in wild fish populations with the aim of translating these ecological patterns into feeding and health-management strategies for aquaculture or stock enhancement.

The Hongshui River is an important tributary of the Pearl River Basin in southern China and has experienced extensive hydropower development during recent decades. Among the hydraulic infrastructures constructed within the basin, the Datengxia Water Conservancy Project is one of the largest and most influential. Reservoir impoundment associated with this project may contribute to ecological differentiation between upstream and downstream habitats, potentially affecting resource utilization patterns of resident fish species. *Hemibagrus guttatus* (*H. guttatus*) is an economically important benthic freshwater fish that primarily feeds on benthic invertebrates and small aquatic animals and is therefore closely associated with local trophic conditions [15]. *H. guttatus* is distributed mainly in river systems of southern China and represents an important indigenous freshwater fishery resource in this region. In addition to its economic value, the species is of interest for conservation and stock-enhancement programs because natural populations are closely associated with riverine habitats that are increasingly influenced by hydropower development and other anthropogenic disturbances. Therefore, understanding its trophic ecology, gut microbial characteristics, and nutritional status under contrasting river habitats has both ecological and applied significance. Owing to its close association with local food resources, *H. guttatus* provides a suitable model for examining whether naturally occurring dietary and microbial differences among source populations should be considered in artificial propagation, nutritional conditioning, and stock enhancement.

In the present study, upstream and downstream populations of *H. guttatus* were collected from habitats adjacent to the Datengxia Water Conservancy Project. Dietary composition was characterized using mitochondrial 12S rRNA and COI metabarcoding, intestinal bacterial communities were examined using 16S rRNA gene sequencing, and muscle moisture, crude protein, and crude lipid contents were determined. We asked whether fish from the two habitats differed consistently in natural food use, gut microbial composition, and muscle nutritional traits. We further considered the potential relevance of these population-specific profiles to future nutritional and microbiota-oriented research in *H. guttatus* aquaculture and stock enhancement.

## 2. Materials and Methods

### 2.1. Sample Collection

**Figure 1:**
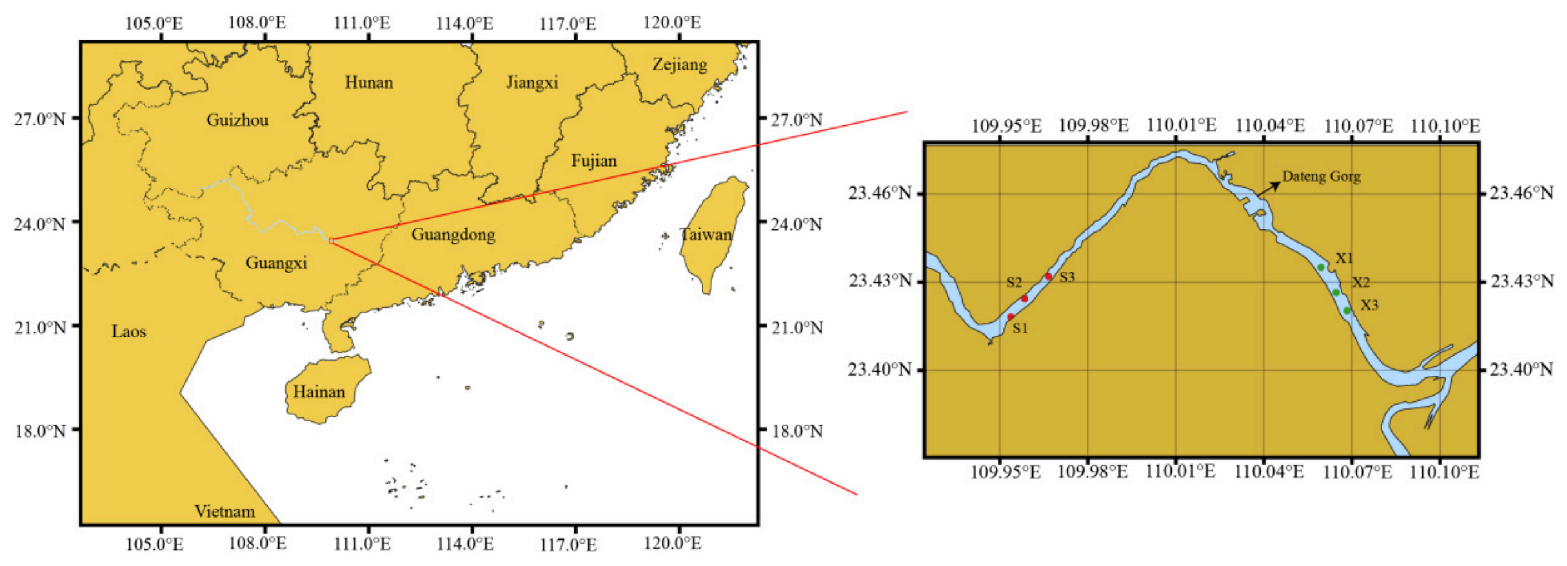
Geographic location of the sampling sites for *H. guttatus* in the Hongshui River, China. The left panel shows the location of the study area within southern China, and the right panel shows the upstream (S1–S3) and downstream (X1–X3) sampling sites located on either side of the Datengxia Water Conservancy Project. Two fish were collected from each sampling site.

Fish samples were collected in September 2025 from two locations situated upstream and downstream of the Datengxia Water Conservancy Project in the Hongshui River Basin, southern China. September was selected as a hydrologically relevant sampling period because it generally corresponds to the late flood season and the transition toward post-flood reservoir regulation in the Datengxia system. During this period, differences between upstream impoundment and downstream regulated discharge may be particularly relevant to habitat conditions. The September survey therefore provides a seasonal snapshot of upstream–downstream biological variation under a dam-regulated hydrological regime rather than a representation of year-round conditions. The sampling sites represented an impounded habitat upstream of the dam and a flow-regulated habitat downstream. The geographic coordinates were 23.4244° N, 109.9584° E for the upstream site and 23.4265° N, 110.0643° E for the downstream site (Figure 1).

A total of 12 wild *H. guttatus* individuals were collected, with two individuals obtained from each of the three upstream sampling sites (S1–S3) and two individuals from each of the three downstream sampling sites (X1–X3), resulting in six individuals per habitat. Fish were captured using gill nets and transported to the laboratory on ice for sample processing. Body length, body weight, sex, and age were not systematically recorded during field sampling. The gastrointestinal tract of each fish was dissected, and stomach contents were collected for dietary metabarcoding analyses. For gut microbiota analysis, intestinal tissues were gently rinsed with sterile phosphate-buffered saline (PBS) to remove residual intestinal contents before sampling. Intestinal tissue samples were then collected for microbial community analysis. In addition, dorsal muscle tissues were sampled for nutritional composition analysis. All samples were immediately frozen in liquid nitrogen and stored at −80 °C until DNA extraction and subsequent analyses.

### 2.2. DNA Extraction

To distinguish dietary DNA from resident intestinal microbiota, stomach-content samples and intestinal tissue samples were processed separately. Total genomic DNA from stomach contents was extracted using the DNeasy PowerSoil Kit (Qiagen, Hilden, Germany) following the manufacturer’s instructions for dietary metabarcoding analysis. For gut microbiota analysis, microbial genomic DNA associated with intestinal tissues was extracted using the same extraction protocol. DNA concentration and purity were assessed using a NanoDrop 2000 spectrophotometer (Thermo Scientific, Waltham, MA, USA), and DNA integrity was further verified by 1.0% agarose gel electrophoresis.

### 2.3. 12S rRNA Gene Amplification and Sequencing for Dietary Analysis

The dietary composition of *H. guttatus* was characterized using metabarcoding of the mitochondrial 12S rRNA gene amplified from stomach-content DNA. A hypervariable region of the 12S rRNA gene was amplified using the universal primer pair MiFish-U (forward: GTCGGTAAAACTCGTGCCAGC; reverse: CATAGTGGGGTATCTAATCCCAGTTTG) [16]. Sequencing adapters were attached to the 5′ ends of both primers prior to PCR amplification. To reduce potential amplification bias, each sample was amplified in triplicate, and the resulting products were pooled prior to library preparation.

PCR reactions were carried out in a total volume of 25 μL, containing 12.5 μL of 2× Taq PCR Master Mix (Takara, Dalian, China), 1.0 μL of each primer (10 μM), 2.0 μL of template DNA, and nuclease-free water to the final volume. Amplification was conducted under the following conditions: an initial denaturation at 95 °C for 3 min; 35 cycles of denaturation at 95 °C for 30 s, annealing at 60 °C for 30 s, and extension at 72 °C for 45 s; followed by a final extension at 72 °C for 10 min.

Amplification products were examined by agarose gel electrophoresis and then purified, quantified, and pooled in equimolar concentrations for library construction. After quality assessment, the libraries were sequenced on an Illumina NovaSeq 6000 platform (Illumina, San Diego, CA, USA) using a paired-end sequencing strategy.

### 2.4. COI Gene Amplification and Sequencing for Dietary Analysis

To obtain a broader representation of dietary resources, particularly invertebrate prey, the mitochondrial cytochrome c oxidase subunit I (COI) gene was amplified from stomach-content DNA. A fragment of the COI gene was amplified using the universal metazoan primer pair mlCOIintF (GGWACWGGWTGAACWGTWTAYCCYCC) and jgHCO2198 (TANACYTCNGGRTGNCCRAARAAYCA) [17]. Sequencing adapters were attached to the 5′ ends of both primers. PCR amplification, product purification, quantification, library construction, and sequencing were performed as described in Section 2.3, except that the annealing temperature was set to 48 °C. Each sample was amplified in triplicate before pooling for library preparation.

### 2.5. 16S rRNA Gene Amplification and Sequencing for Gut Microbiota Analysis

To characterize intestinal microbial communities of *H. guttatus*, the V3–V4 hypervariable region of the bacterial 16S rRNA gene was amplified from intestinal tissue-derived DNA using the universal bacterial primers pair 338F (ACTCCTACGGGAGGCAGCA) 806R (GGACTACHVGGGTWTCTAAT) [18]. Sequencing adapters were added to the 5′ ends of the primers. PCR amplification, product purification, quantification, library construction, and sequencing were performed as described in Section 2.3. Each sample was amplified in triplicate to minimize amplification bias.

### 2.6. Bioinformatics Analysis

Raw sequencing reads obtained from 12S, COI, and 16S rRNA gene sequencing were quality-filtered using Trimmomatic v0.33 [19]. Primer sequences and adapters were subsequently identified and removed using Cutadapt v1.9.1o [20] obtain clean reads. Sequence denoising, paired-end merging, and chimera removal were conducted using the DADA2 plugin [21] implemented in QIIME2 v2020.6 [22], resulting in high-quality non-chimeric reads for downstream analyses.

Sequencing quality was evaluated based on sequence counts and read quality across different processing stages, including raw reads, clean reads, denoised reads, merged reads, and non-chimeric reads. Taxonomic assignment of 12S and COI feature sequences was performed against the NCBI database using a naïve Bayes classifier [23] combined with sequence alignment methods. Taxonomic assignment of 16S rRNA gene sequences was performed against the SILVA database (release 138) [24] using a naïve Bayes classifier implemented in QIIME2 [22]. Taxonomic composition at different classification levels, including phylum, class, order, family, genus, and species, was summarized for downstream ecological and microbial community analyses.

### 2.7. Muscle Nutritional Composition Analysis

Dorsal muscle samples collected from *H. guttatus* were used for nutritional composition analysis. Muscle samples were freeze-dried using a lyophilizer (SJIA-10N-50A, Ningbo Shuangjia Instrument Co., Ltd., Ningbo, China) prior to analysis. Moisture content was determined gravimetrically based on weight loss after freeze-drying. Crude lipid content was determined by Soxhlet [25] extraction using diethyl ether as the extraction solvent with a SoxROC extraction system (OPSIS, Furulund, Sweden), and crude protein content was determined by the Kjeldahl [26] method using an automatic Kjeldahl analyzer (KD310, OPSIS, Furulund, Sweden). These analyses were performed following internationally recognized analytical principles described in the Official Methods of Analysis of AOAC INTERNATIONAL [27], with instrument operation conducted according to the manufacturers’ instructions.

### 2.8. Statistical Analysis

Alpha diversity indices, including Shannon, Chao1, and Simpson indices, were calculated to evaluate differences in dietary composition and intestinal microbial diversity between upstream and downstream populations of *H. guttatus*. Normality of each variable was assessed using the Shapiro–Wilk test, and homogeneity of variances was evaluated using Levene’s test. Variables satisfying both assumptions were compared between upstream and downstream groups using Student’s *t*-test. Welch’s *t*-test was applied when the assumption of equal variances was violated, whereas the Mann–Whitney U test was used when the normality assumption was not satisfied. Statistical significance was set at *p* < 0.05. Beta diversity analyses were performed based on Bray–Curtis [28] distance matrices, and principal coordinate analysis (PCoA) was conducted to visualize community dissimilarities among samples. Permutational multivariate analysis of variance (PERMANOVA) [29] was used to evaluate significant differences in dietary composition and intestinal microbial community structure between habitats. Homogeneity of multivariate dispersion between upstream and downstream groups was further evaluated using permutational analysis of multivariate dispersions (PERMDISP) based on the same Bray–Curtis distance matrices. Distances of individual samples to their respective group centroids were calculated, and differences in multivariate dispersion between groups were assessed by permutation tests. PERMDISP was used to determine whether the PERMANOVA results could potentially be influenced by heterogeneity in within-group dispersion.

Relative abundance analyses at different taxonomic levels were conducted to characterize variations in dietary composition and intestinal microbial communities between upstream and downstream populations. Differential taxa between groups were identified using linear discriminant analysis effect size (LEfSe) [30]. Muscle nutritional parameters, including moisture, crude lipid, and crude protein contents, were compared between groups using independent-sample *t*-tests. Statistical analyses were conducted using QIIME2 [22], R software (version 4.2.3), and SPSS software (version 30). Differences were considered statistically significant at *p* < 0.05.

## 3. Results

### 3.1. Sequencing Data Overview

High-throughput sequencing generated a total of 2,878,733 raw reads from the stomach-content 12S rRNA dataset and 1,919,322 raw reads from the COI dataset across all samples. After quality filtering, primer trimming, sequence denoising, paired-end merging, and chimera removal, a total of 2,721,808 and 1,733,737 high-quality non-chimeric reads were retained for downstream analyses in the 12S and COI datasets, respectively. The average numbers of non-chimeric reads per sample were 226,817 for the 12S dataset and 144,478 for the COI dataset. Rarefaction curves for all dietary samples gradually approached saturation, indicating that the sequencing depth was sufficient to capture the majority of prey diversity present in stomach contents (Figure S1). In addition, Good’s coverage values exceeded 99.98% across all samples, suggesting adequate sequencing coverage for subsequent ecological analyses.

For intestinal microbiota analysis, a total of 473,155 raw reads were generated from the 16S rRNA dataset. After sequence denoising using the DADA2 algorithm, 445,122 high-quality non-chimeric reads and 4493 amplicon sequence variants (ASVs) were obtained for downstream microbial community analyses.

Detailed sequencing statistics for all datasets, including raw reads, clean reads, denoised reads, merged reads, and non-chimeric reads, are summarized in Table S1.

### 3.2. Dietary Composition Based on Stomach-Content 12S Metabarcoding

#### 3.2.1. Diversity and Community Structure of Dietary Composition

**Figure 2:**
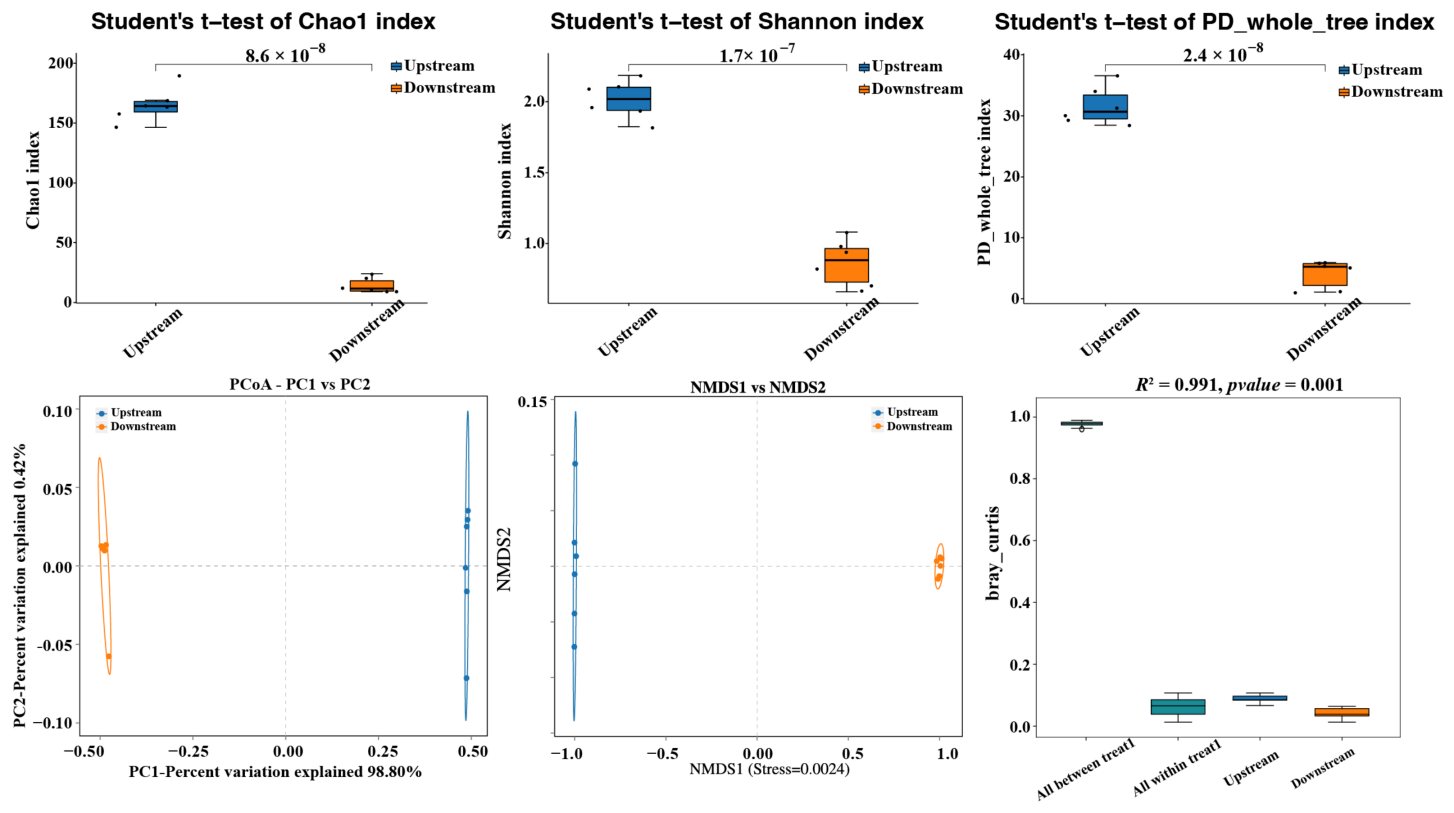
Alpha and beta diversity of prey communities identified by 12S rRNA metabarcoding in the stomach contents of upstream and downstream *H. guttatus*. The upper panels show comparisons of the Chao1, Shannon, and PD whole tree indices between the two groups. The lower panels present principal coordinate analysis (PCoA), non-metric multidimensional scaling (NMDS), and analysis of similarities (ANOSIM) based on Bray–Curtis distances. *p* values for comparisons between the upstream and downstream groups are shown directly in each panel, with *p* < 0.05 considered statistically significant.

Alpha diversity analysis revealed significant differences in dietary diversity between upstream and downstream populations of *H. guttatus* based on stomach-content 12S metabarcoding (Figure 2 and Figure S2). The upstream group exhibited significantly higher ACE, Chao1, Shannon, Simpson, and phylogenetic diversity (PD whole tree) indices than the downstream group (*p* < 0.05 for all comparisons). These results indicate substantially greater prey richness and dietary diversity in upstream individuals, whereas downstream fish exhibited relatively simplified dietary composition. In particular, the Shannon and Chao1 indices indicated substantially greater prey richness and dietary diversity in upstream individuals, whereas downstream fish exhibited relatively simplified dietary composition.

Beta diversity analysis based on Bray–Curtis dissimilarity further revealed clear separation between upstream and downstream dietary communities (Figure 2). Principal coordinate analysis (PCoA) showed that samples from the two habitats formed distinct clusters along the first principal coordinate axis, which explained 98.98% of the total variation. Similarly, non-metric multidimensional scaling (NMDS) analysis demonstrated strong segregation between upstream and downstream groups with a low stress value (Stress = 0.002), indicating robust ordination reliability. PERMANOVA analysis indicated significant differentiation in 12S-derived dietary composition between upstream and downstream populations (R^2^ = 0.992, *p* = 0.001). However, PERMDISP analysis also detected a modest but significant difference in multivariate dispersion between the two groups (F = 3.74, *p* = 0.046), with a higher mean distance to the group centroid in downstream samples (0.0243) than in upstream samples (0.0164). Thus, although the ordination analyses showed pronounced separation between upstream and downstream dietary communities, the PERMANOVA result may partly reflect differences in within-group dispersion and should be interpreted with caution. In addition, Bray–Curtis dissimilarity within groups was substantially lower than that between groups, suggesting strong habitat-associated differentiation in dietary composition.

#### 3.2.2. Taxonomic Composition and Differential Prey Taxa

**Figure 3:**
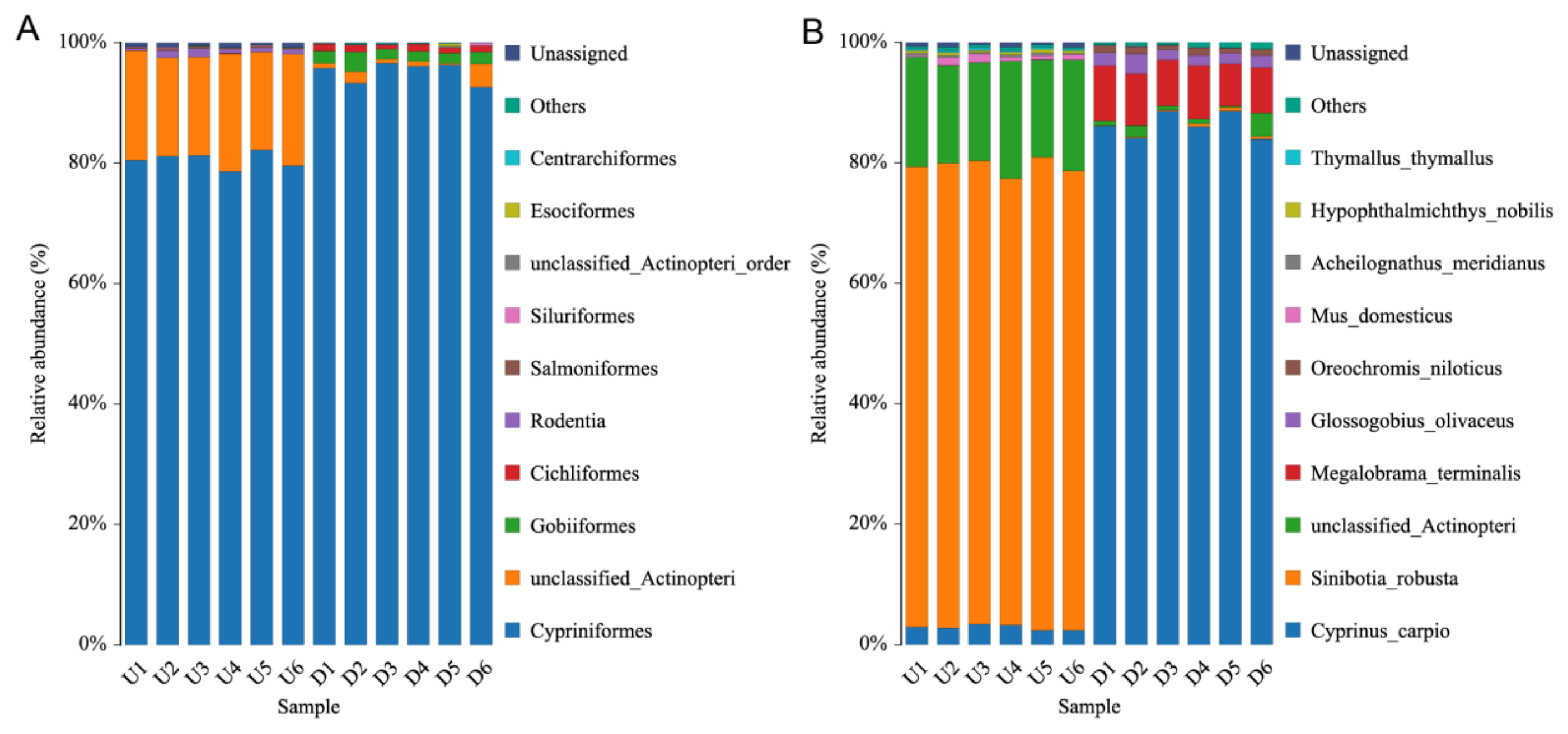
Relative abundance of the dominant prey taxa identified by 12S rRNA metabarcoding in the stomach contents of upstream and downstream *H. guttatus*. (**A**) Order-level taxonomic composition. (**B**) Species-level taxonomic composition. Only the most abundant taxa are shown, while the remaining low-abundance taxa are grouped as “Others”.

Taxonomic composition analysis based on 12S metabarcoding further demonstrated pronounced differences in dietary structure between upstream and downstream populations of *H. guttatus* (Figure 3A). At the order level, Cypriniformes was the most abundant prey group in all samples. Its relative abundance ranged from 78.4% to 82.1% in upstream individuals and from 92.1% to 95.7% in downstream individuals. In contrast, unclassified Actinopterii accounted for a larger proportion of the upstream diet. Gobiiformes and Cichliformes were detected at low abundances overall but were more frequently observed in downstream samples. These results indicate that although Cypriniformes dominated the dietary composition throughout the study area, differences between upstream and downstream fish were reflected in the occurrence and relative abundance of less common prey groups.

At the species level, dietary composition differed markedly between upstream and downstream populations (Figure 3B). *Sinibotia robusta* was the dominant prey species in upstream fish, accounting for approximately 75–80% of total reads, whereas Cyprinus carpio represented the major dietary component in downstream individuals, contributing 84–89% of dietary sequences. In addition, Megalobrama terminalis and *Glossogobius olivaceus* occurred at higher relative abundances in downstream samples. Upstream fish contained a greater proportion of unclassified Actinopterii (approximately 15–20%), while this group accounted for less than 5% of reads in downstream samples. Overall, the dominant prey species shifted from *Sinibotia robusta* upstream to Cyprinus carpio downstream.

**Figure 4:**
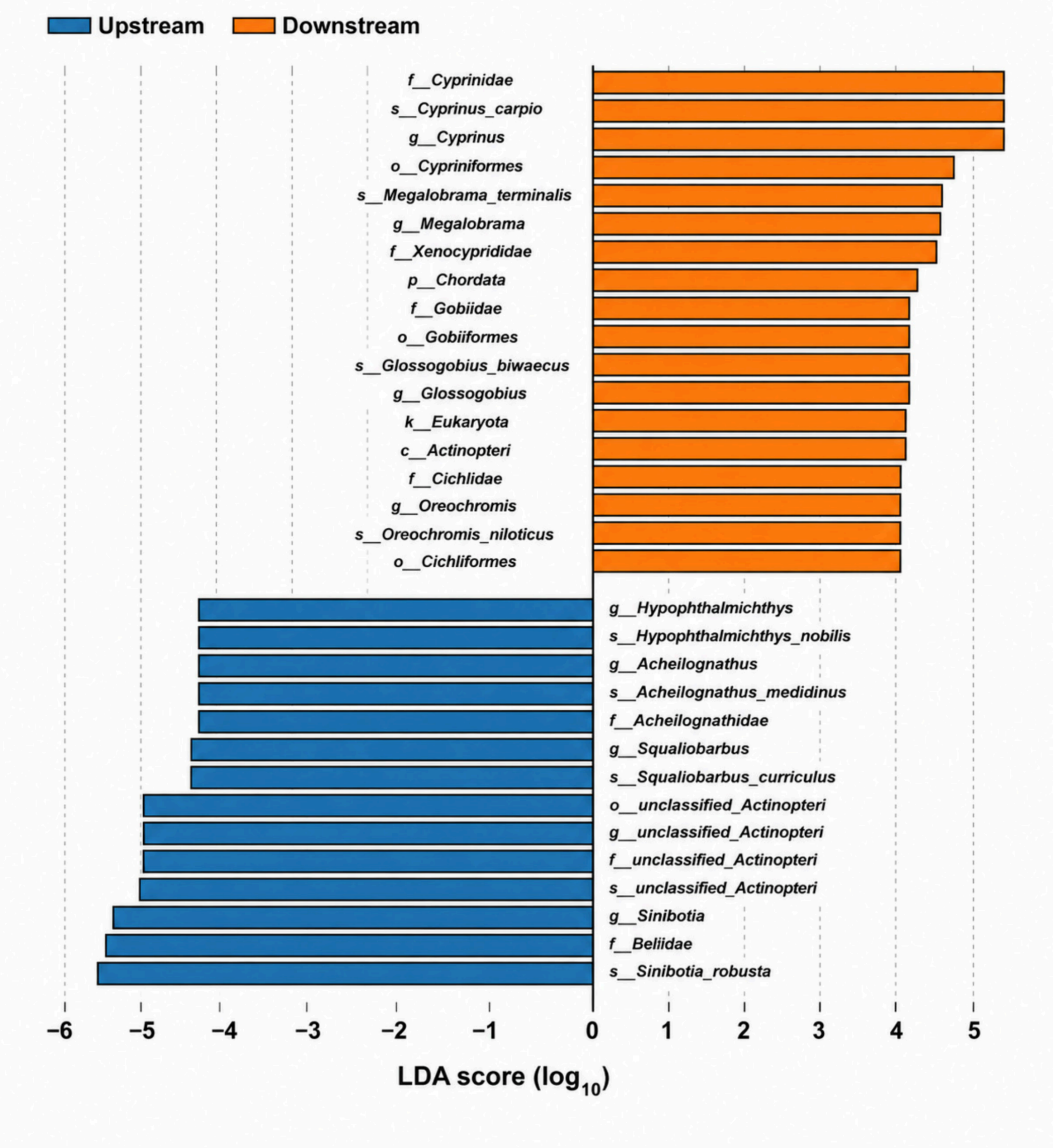
Linear discriminant analysis effect size (LEfSe) identifying differentially abundant prey taxa between upstream and downstream *H. guttatus* based on 12S rRNA metabarcoding. Taxa with positive LDA scores are enriched in the downstream group, whereas taxa with negative LDA scores are enriched in the upstream group. Only taxa with an LDA score > 4.0 are shown.

LEfSe analysis revealed clear differences in indicator taxa between habitats (Figure 4). *Sinibotia robusta*, *Squaliobarbus curriculus*, and *Hypophthalmichthys nobilis* were enriched in upstream samples, whereas *Cyprinus carpio*, Megalobrama terminalis, *Glossogobius olivaceus*, and *Oreochromis niloticus* were enriched in downstream samples. Most discriminative taxa had LDA scores above 4.0.

### 3.3. COI-Based Dietary Composition

#### 3.3.1. Diversity and Community Structure of COI-Derived Dietary Taxa

**Figure 5:**
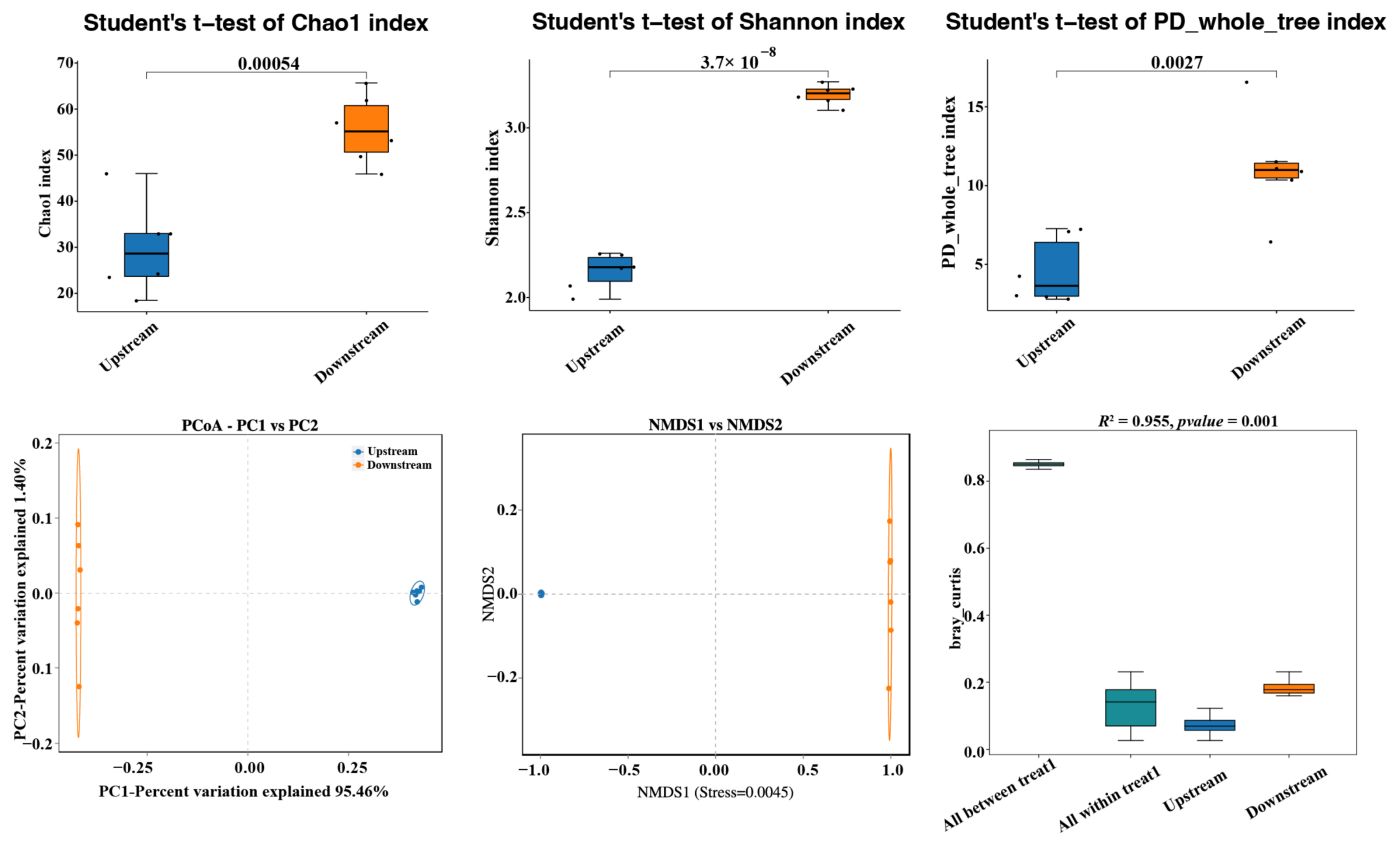
Alpha and beta diversity of prey communities identified by COI metabarcoding in the stomach contents of upstream and downstream *H. guttatus*. The upper panels show comparisons of the Chao1, Shannon, and PD whole tree indices between the two groups. The lower panels present principal coordinate analysis (PCoA), non-metric multidimensional scaling (NMDS), and analysis of similarities (ANOSIM) based on Bray–Curtis distances. *p* values for comparisons between the upstream and downstream groups are shown directly in each panel, with *p* < 0.05 considered statistically significant.

COI-based alpha diversity differed between upstream and downstream populations of *H. guttatus* (Figure 5). Downstream samples exhibited significantly higher Chao1, Shannon, Simpson, and PD whole tree indices than upstream samples (*p* < 0.05), whereas no significant difference was detected in the ACE index (*p* > 0.05; Figure S2). These results indicate greater diversity of COI-derived dietary taxa in downstream fish for most alpha-diversity metrics.

COI-based beta diversity differed between upstream and downstream populations (Figure 5). In the PCoA ordination, samples from the two habitats formed separate clusters, and the first axis explained 95.46% of the variation. PERMANOVA analysis indicated significant differentiation in COI-derived dietary composition between upstream and downstream populations (R^2^ = 0.955, *p* = 0.001). PERMDISP analysis also revealed significant heterogeneity in multivariate dispersion between the two groups (F = 17.01, *p* = 0.002), with a greater mean distance to the group centroid in downstream samples (0.0564) than in upstream samples (0.0319). Therefore, the PERMANOVA result may reflect both differences in community location and unequal within-group dispersion, and the magnitude of the upstream–downstream differentiation should be interpreted cautiously. Pairwise Bray–Curtis distances were higher between habitats than within habitats. A similar separation was observed in the NMDS analysis (stress = 0.0045).

#### 3.3.2. Taxonomic Composition and Differential Prey Taxa

**Figure 6:**
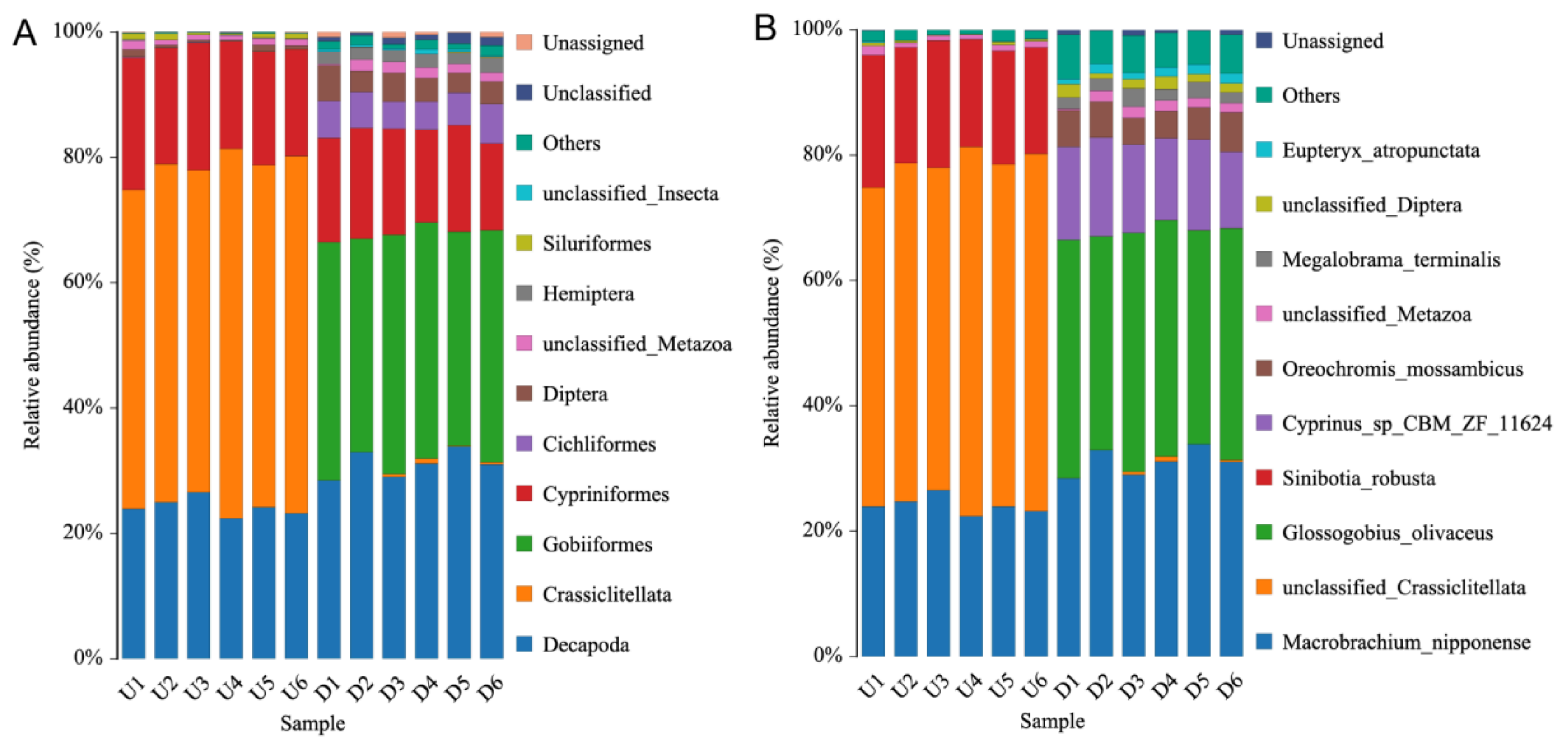
Relative abundance of the dominant prey taxa identified by COI metabarcoding in the stomach contents of upstream and downstream *H. guttatus*. (**A**) Order-level taxonomic composition. (**B**) Species-level taxonomic composition. Only the most abundant taxa are shown, while the remaining low-abundance taxa are grouped as “Others”.

Taxonomic composition differed between upstream and downstream samples at the order level (Figure 6A). Crassiclitellata was the dominant taxon in upstream individuals, accounting for more than half of the detected prey items, while Decapoda and Cypriniformes represented the second and third most abundant groups. In downstream samples, Decapoda and Gobiiformes comprised a larger proportion of the diet, whereas the contribution of Crassiclitellata was substantially lower. Cichliformes and Diptera were also more frequently detected in downstream individuals. Patterns observed at the species level were consistent with those detected at higher taxonomic ranks (Figure 6B). Upstream diets were characterized by high proportions of unclassified Crassiclitellata together with *Macrobrachium nipponense* and *Sinibotia robusta*. In contrast, downstream samples contained greater proportions of *Glossogobius olivaceus* and *Macrobrachium nipponense*, accompanied by moderate contributions from *Cyprinus* sp. CBM_ZF_11624 and Oreochromis mossambicus. Other taxa, including *Megalobrama terminalis*, unclassified Diptera and *Eupteryx atropunctata*, occurred at relatively low abundances across downstream samples.

**Figure 7:**
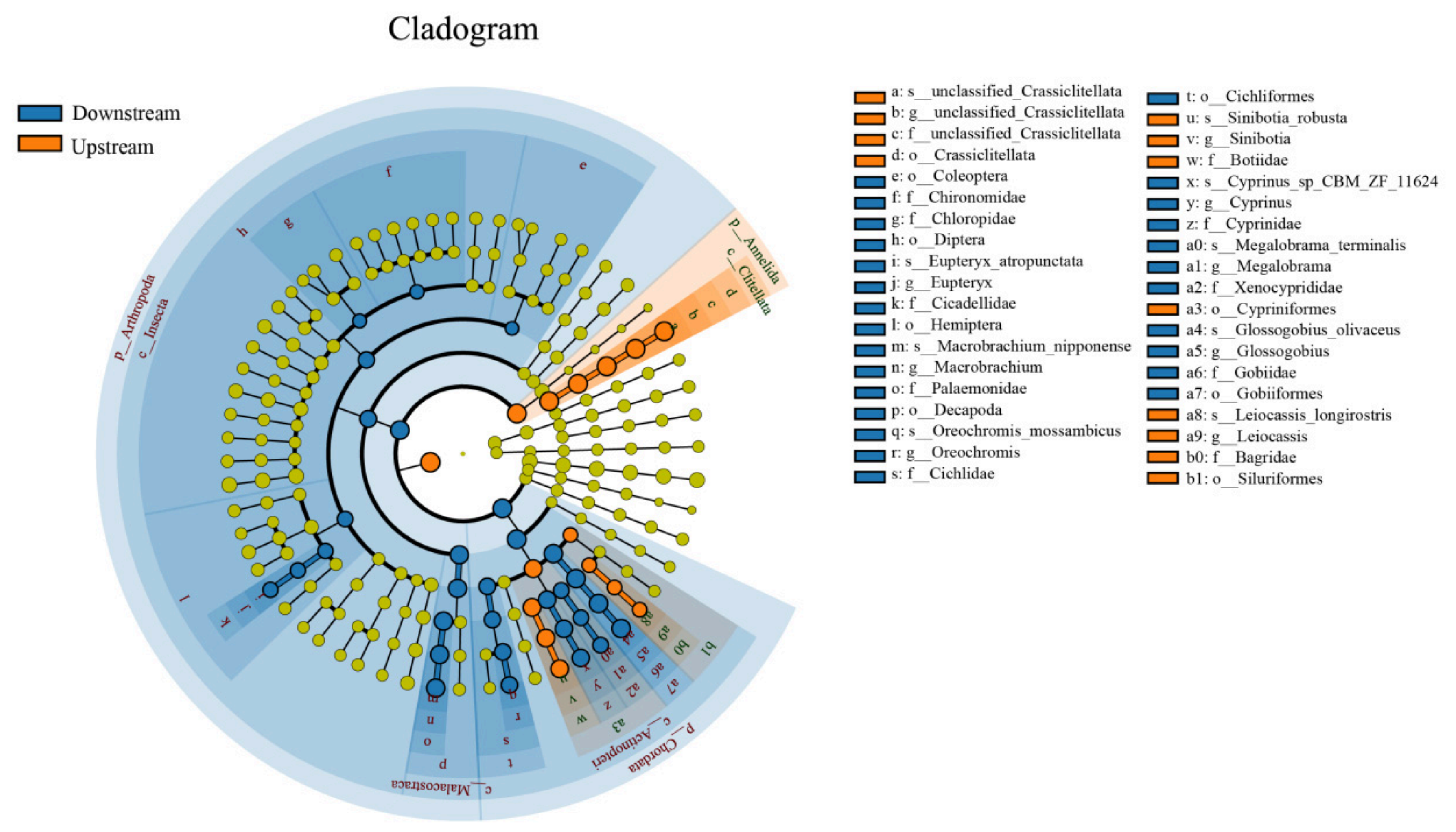
Linear discriminant analysis effect size (LEfSe) identifying differentially abundant prey taxa between upstream and downstream *H. guttatus* based on COI metabarcoding. The cladogram illustrates the phylogenetic distribution of significantly enriched taxa in each group. The colored dots represent taxonomic nodes showing significant differential enrichment, with blue and orange indicating taxa enriched in the upstream and downstream groups, respectively.

LEfSe analysis identified clear taxonomic differences between habitats (Figure 7). Upstream samples were characterized by enrichment of Crassiclitellata-related taxa, together with Diptera, Hemiptera and Decapoda, including *Macrobrachium nipponense*. Downstream samples showed higher abundances of several fish-associated taxa, including *Glossogobius olivaceus*, *Sinibotia robusta*, *Cyprinus* sp., *Megalobrama terminalis*, *Oreochromis mossambicus* and *Leiocassis longirostris*, as well as the corresponding higher taxonomic groups Gobiiformes, Cypriniformes and Siluriformes.

### 3.4. Gut Bacterial Community Characteristics Based on 16S rRNA Sequencing

#### 3.4.1. Alpha Diversity of Gut Microbiota

**Figure 8:**
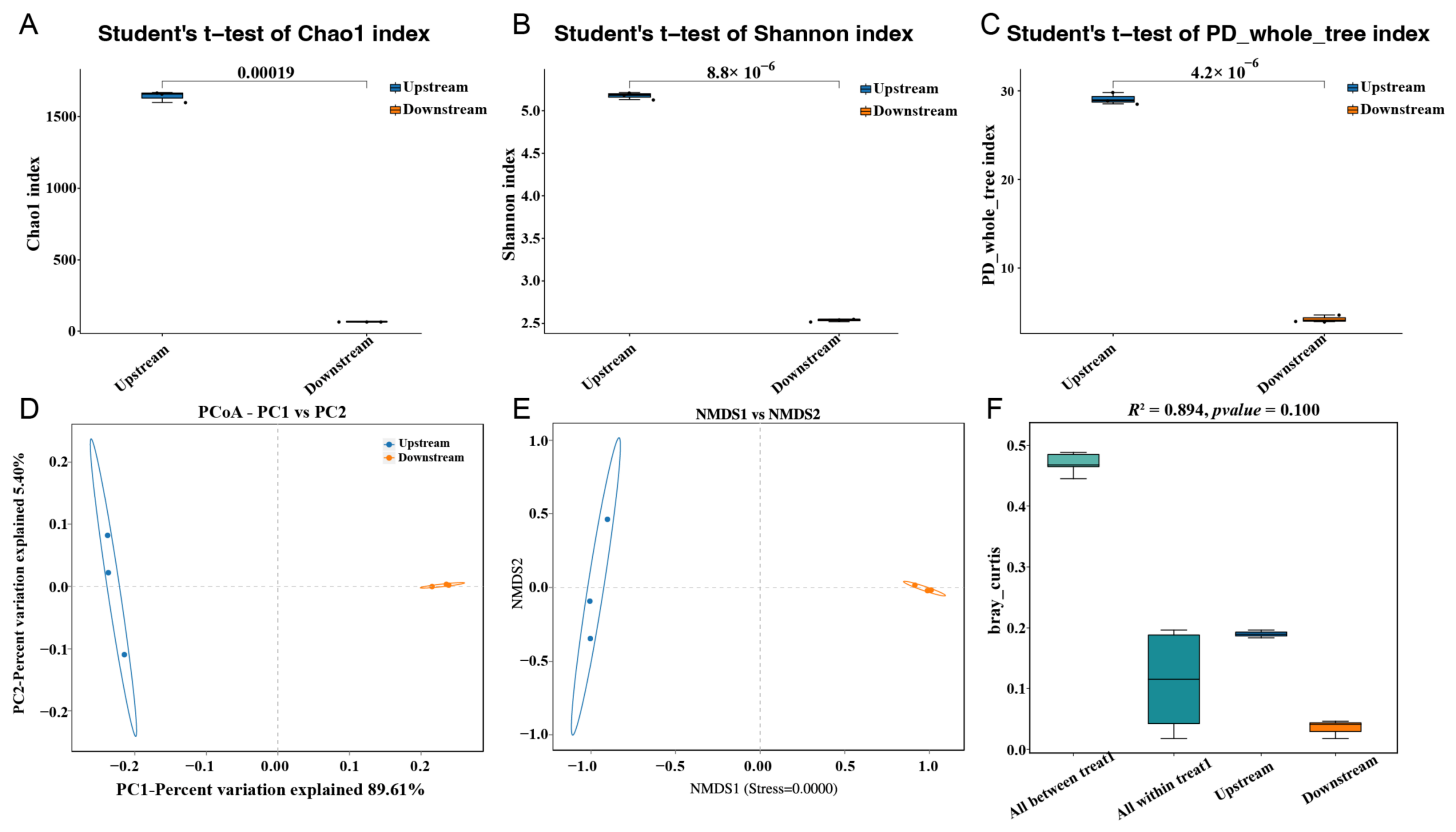
Alpha and beta diversity of intestinal bacterial communities in upstream and downstream *H. guttatus* based on 16S rRNA gene sequencing. (**A**) Chao1 index; (**B**) Shannon index; (**C**) PD whole tree index; (**D**) principal coordinate analysis (PCoA); (**E**) non-metric multidimensional scaling (NMDS); and (**F**) analysis of similarities (ANOSIM) based on Bray–Curtis distances. *p* values for comparisons between the upstream and downstream groups are shown directly in each panel, with *p* < 0.05 considered statistically significant.

Alpha diversity differed significantly between upstream and downstream populations (Figure 8A–C). ACE, Chao1, PD whole tree, Shannon, and Simpson indices were all significantly higher in the upstream group than in the downstream group (Student’s *t*-test, *p* < 0.001 for all comparisons). Across all alpha-diversity metrics, upstream individuals consistently exhibited higher values, indicating greater richness and diversity of gut microbial communities in the upstream population.

#### 3.4.2. Beta Diversity of Gut Microbiota

Beta diversity analysis showed differences in gut microbial community composition between upstream and downstream populations (Figure 8D–F). In the PCoA ordination based on Bray–Curtis distances, samples from the two groups formed separate clusters along the first axis, which explained 89.61% of the total variation (Figure 8D). A similar pattern was observed in the NMDS analysis, where upstream and downstream samples occupied distinct positions in the ordination space (Figure 8E). The NMDS stress value was close to zero, indicating a good representation of community relationships. PERMANOVA analysis showed that sampling location accounted for 89.4% of the variation in microbial community composition (R^2^ = 0.894), although the difference was not statistically significant (*p* = 0.100) (Figure 8F). PERMDISP analysis likewise did not detect a statistically significant difference in multivariate dispersion between the upstream and downstream groups (F = 69.99, *p* = 0.100), although the mean distance to the group centroid was higher in upstream samples (0.0604) than in downstream samples (0.0186). Given the limited number of samples included in the 16S analysis, both the apparent separation in ordination space and the difference in dispersion should be interpreted cautiously as descriptive patterns rather than statistically confirmed group-level differences.

#### 3.4.3. Community Composition of Gut Microbiota

**Figure 9:**
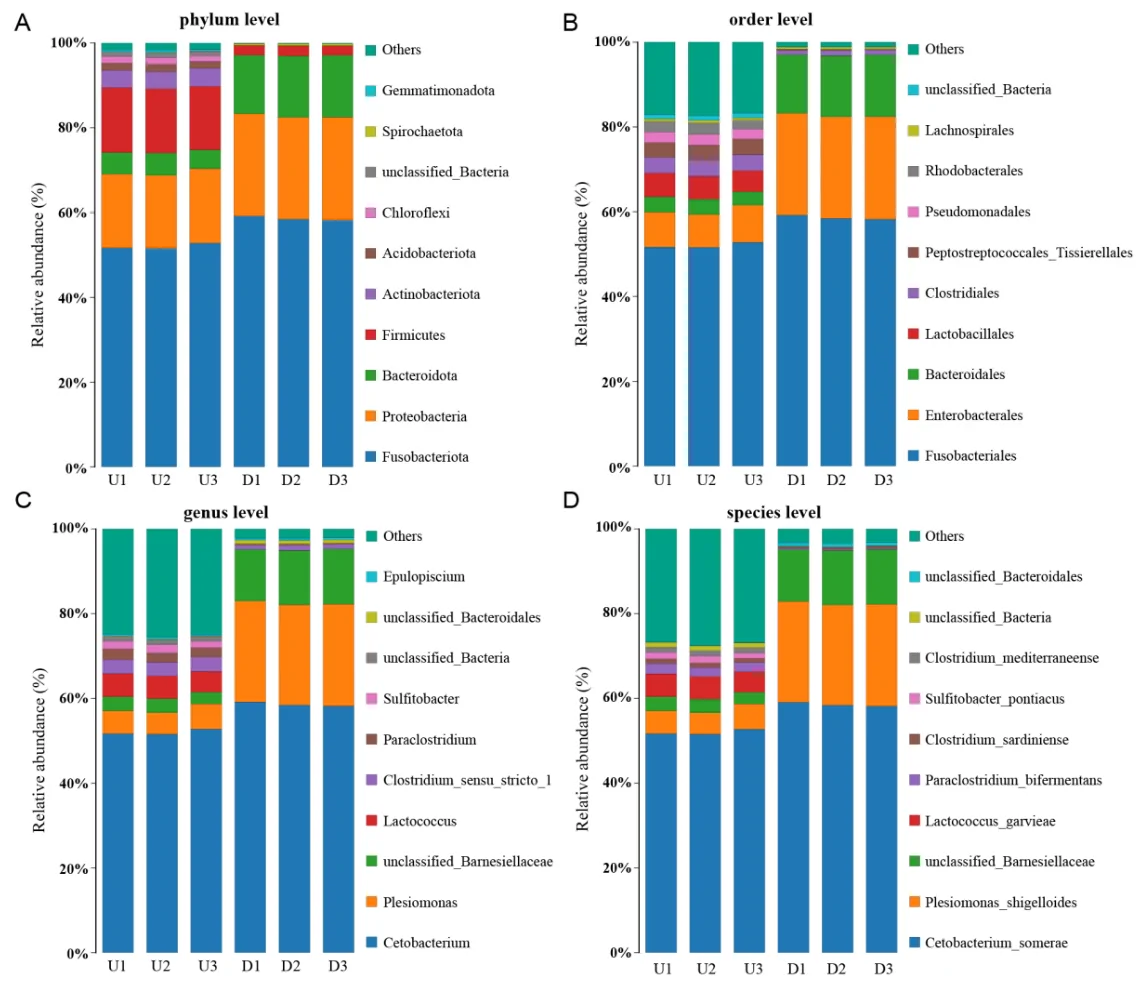
Relative abundance of the dominant intestinal bacterial taxa in upstream and downstream *H. guttatus*. (**A**) Phylum level; (**B**) order level; (**C**) genus level; and (**D**) species level. Only the most abundant taxa are displayed, while the remaining low-abundance taxa are grouped as “Others”.

At the phylum level, Fusobacteriota and Proteobacteria were the dominant bacterial phyla in both upstream and downstream fish (Figure 9A). Their relative abundances, however, differed between groups. Fusobacteriota, Proteobacteria, and Bacteroidota were more abundant in the downstream group, whereas Firmicutes and Actinobacteriota accounted for a larger proportion of the microbiota in upstream fish. At the order level, Fusobacteriales and Enterobacterales represented the major bacterial orders across all samples (Figure 9B). Bacteroidales and Enterobacterales contributed a greater proportion of the community in the downstream group, while Lactobacillales, Clostridiales, and Peptostreptococcales-Tissierellales were comparatively more abundant in the upstream group. At the genus level, Cetobacterium was the predominant taxon in all samples, comprising more than half of the total sequences recovered (Figure 9C). Relative abundances of Plesiomonas and unclassified_Barnesiellaceae were higher in downstream fish, whereas Lactococcus, Clostridium_sensu_stricto_1, and Paraclostridium were more common in upstream individuals. A similar pattern was observed at the species level (Figure 9D). *Cetobacterium*_*somerae* and *Plesiomonas*_*shigelloides* were more abundant in the downstream group, while *Lactococcus*_*garvieae*, *Clostridium*_*sardiniense*, and *Paraclostridium*_*bifermentans* occurred at higher relative abundances in the upstream group.

#### 3.4.4. Differential Microbial Taxa Identified by Lefse

**Figure 10:**
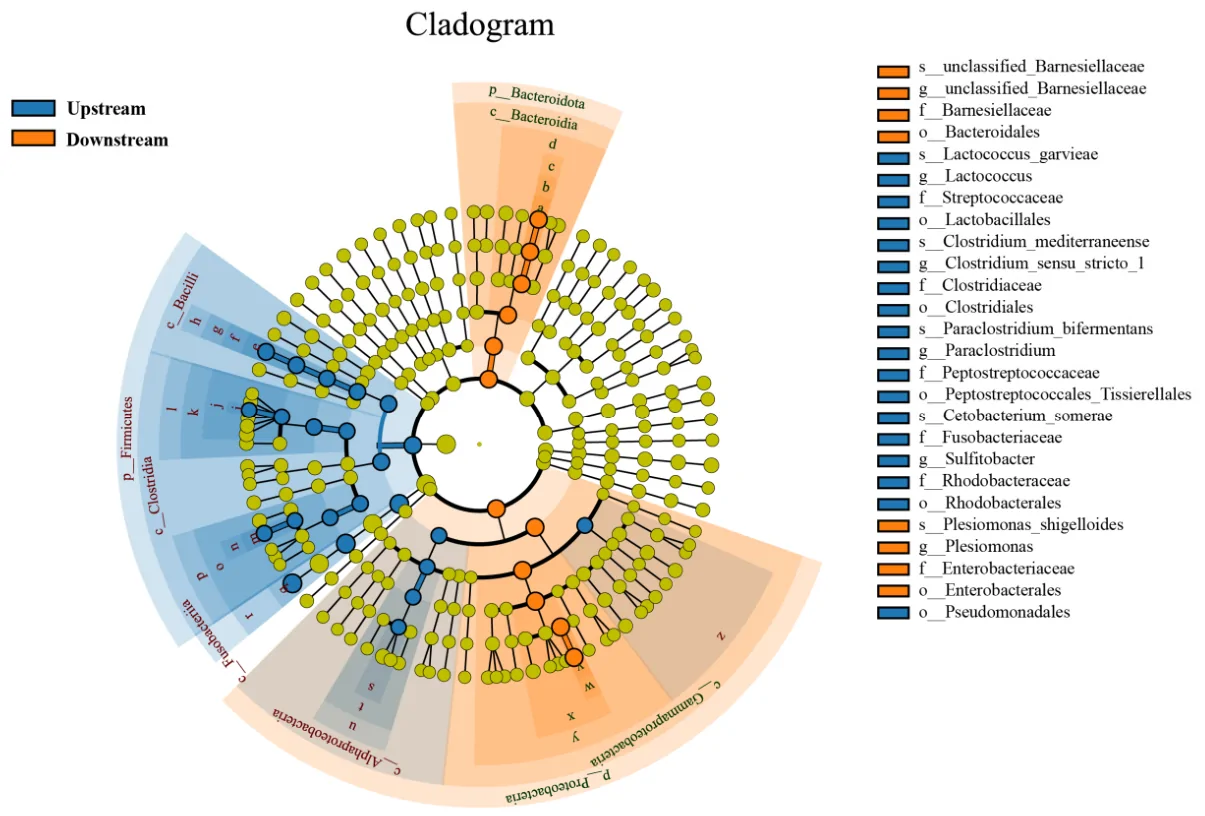
Linear discriminant analysis effect size (LEfSe) cladogram showing differentially abundant intestinal bacterial taxa between upstream and downstream *H. guttatus*. Blue and orange nodes indicate taxa significantly enriched in the upstream and downstream groups, respectively, whereas yellow nodes indicate taxa without significant differences between the two groups. Letters within the cladogram correspond to the differentially abundant taxa listed in the legend on the right.

LEfSe analysis revealed clear differences in the gut microbial taxa between upstream and downstream fish populations (Figure 10). Taxa enriched in upstream samples were mainly assigned to the phylum Firmicutes, particularly members of the classes Bacilli and Clostridia, as well as the orders Lactobacillales and Clostridiales. Several genera and species, including Lactococcus, Lactococcus garvieae, Clostridium sensu stricto 1, and *Paraclostridium bifermentans*, were consistently more abundant in upstream individuals. In contrast, downstream fish were characterized by a higher representation of taxa belonging to Proteobacteria and Bacteroidota. Among these, Gammaproteobacteria, Enterobacterales, and members of the family Barnesiellaceae showed significant enrichment. At finer taxonomic resolution, Plesiomonas and *Plesiomonas shigelloides* were identified as the dominant discriminatory taxa in downstream samples.

#### 3.4.5. Microbial Co-Occurrence Network Analysis

**Figure 11:**
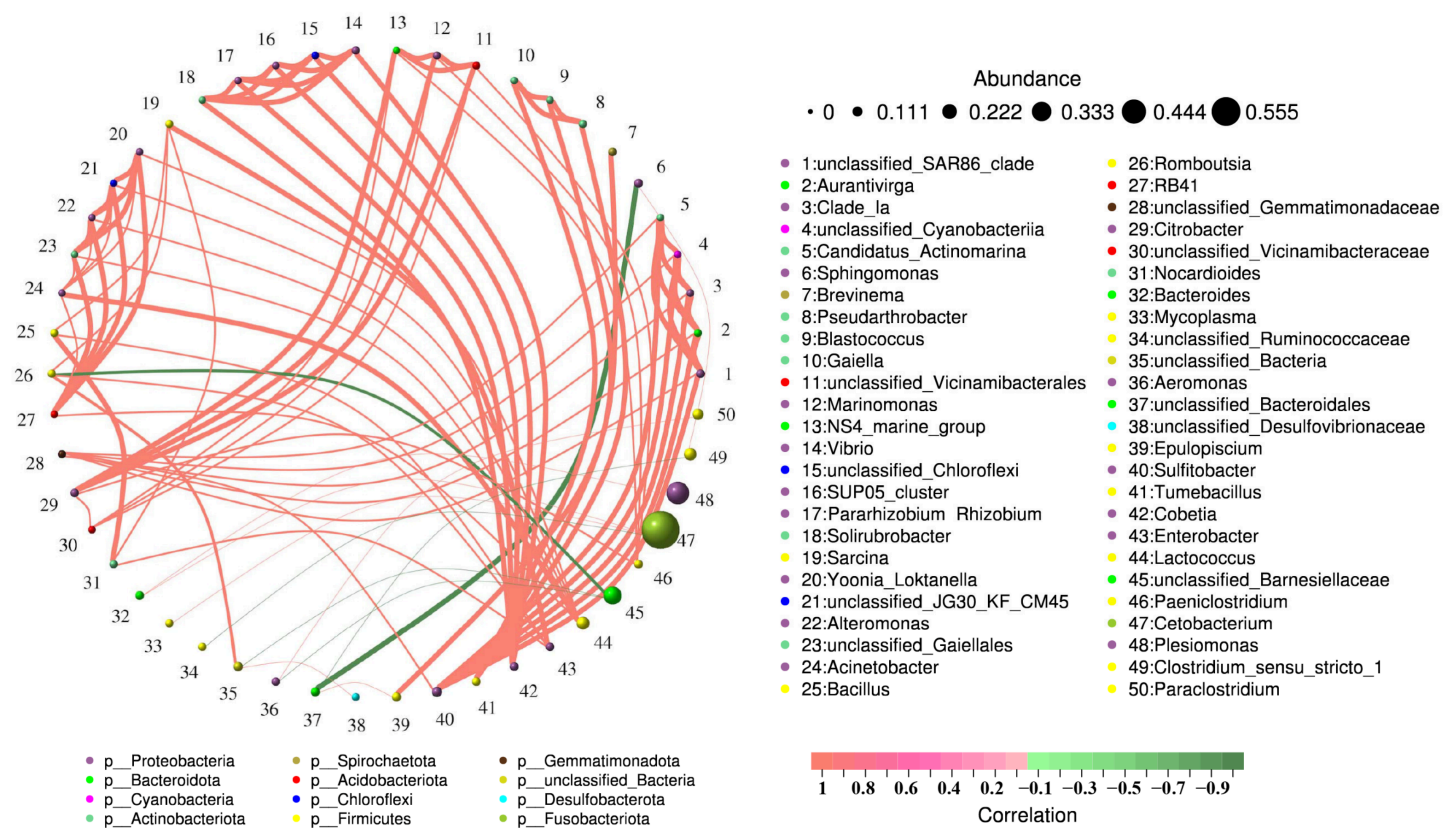
Co-occurrence network of the dominant intestinal bacterial genera in *H. guttatus*. Node size represents the relative abundance of each genus, and node color indicates bacterial phylum. Red and green edges represent positive and negative correlations, respectively, with edge thickness proportional to the correlation strength.

Co-occurrence network analysis showed that the intestinal microbiota of the upstream and downstream groups was composed mainly of members belonging to Proteobacteria, Bacteroidota, Firmicutes, Actinobacteriota, Acidobacteriota, Chloroflexi, and Fusobacteriota (Figure 11). Most connections in the network were positive, indicating that positive associations were more common than negative associations among bacterial taxa. Several abundant genera, including Cetobacterium, Plesiomonas, Lactococcus, Paraclostridium, and Sulfitobacter, were connected with multiple neighboring taxa and displayed relatively high degrees within the network. Among them, Cetobacterium showed numerous positive associations with other bacterial groups. Taxa affiliated with Proteobacteria and Bacteroidota formed several densely connected subnetworks. In contrast, a smaller number of negative correlations were observed among specific taxa distributed across different modules. These positive and negative associations together contributed to the overall network structure. The resulting network exhibited a high level of connectivity, suggesting extensive associations among members of the intestinal bacterial community.

#### 3.4.6. Functional and Phenotypic Prediction Analysis

**Figure 12:**
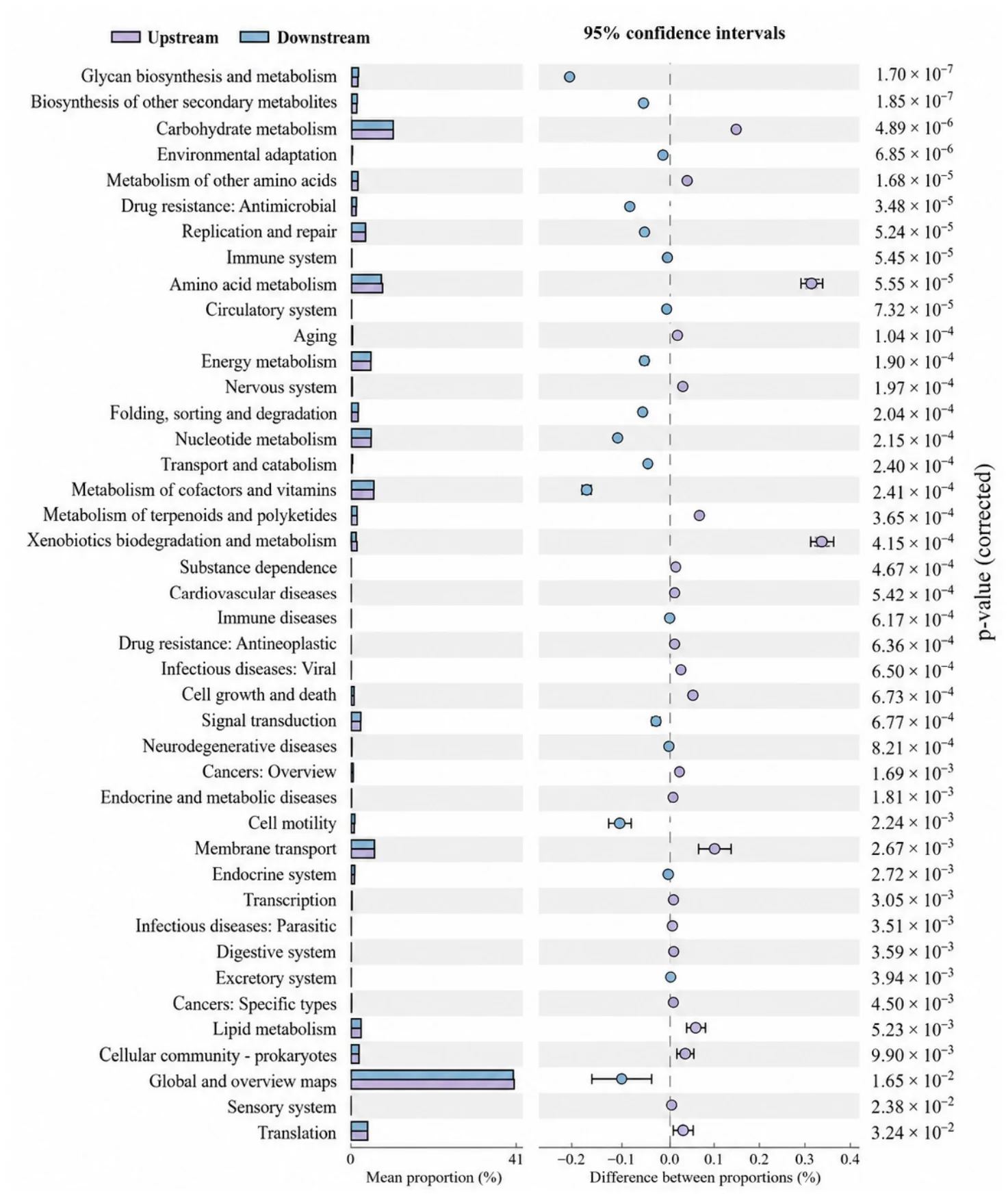
Functional profiles of the intestinal bacterial communities predicted by PICRUSt2. Differentially enriched Kyoto Encyclopedia of Genes and Genomes (KEGG) level 2 pathways between upstream and downstream *H. guttatus* are shown. Bars represent the mean relative abundance of each pathway, and dots indicate the differences between groups with 95% confidence intervals. *p* values for comparisons between the upstream and downstream groups are shown directly in each panel, with *p* < 0.05 considered statistically significant. Purple and blue dots represent the differences in proportions for the upstream and downstream groups, respectively, with horizontal error bars indicating 95% confidence intervals.

PICRUSt2-based functional prediction indicated clear differences in the potential metabolic functions of intestinal bacterial communities between the upstream and downstream groups (Figure 12). Several pathways, including amino acid metabolism, membrane transport, lipid metabolism, translation, cellular community–prokaryotes, and xenobiotics biodegradation and metabolism, were more abundant in the downstream group. In contrast, functions related to glycan biosynthesis and metabolism, biosynthesis of secondary metabolites, replication and repair, nucleotide metabolism, and transport and catabolism showed relatively higher abundances in the upstream group. Among the significantly differentiated pathways, environmental adaptation and xenobiotic degradation were notably enriched in downstream fish. Pathways involved in membrane transport and energy metabolism also tended to be more abundant in this group. These patterns suggest functional shifts in the downstream microbial community, particularly in pathways associated with environmental responses and substrate utilization. The FAPROTAX analysis further revealed differences in ecological functions between the two groups (Figure S3). Functional categories related to aerobic chemoheterotrophy, aromatic compound degradation, hydrocarbon degradation, nitrogen respiration, nitrate respiration, methanol oxidation, methylotrophy, and sulfur oxidation processes were significantly enriched in the downstream group. In addition, functions associated with oil bioremediation, animal parasites or symbionts, and several pathogen-related categories were detected at higher relative abundances in downstream samples. By comparison, fermentation and chemoheterotrophic functions were more prominent in the upstream group.

### 3.5. Nutritional Composition of H. guttatus Muscle

**Figure 13:**
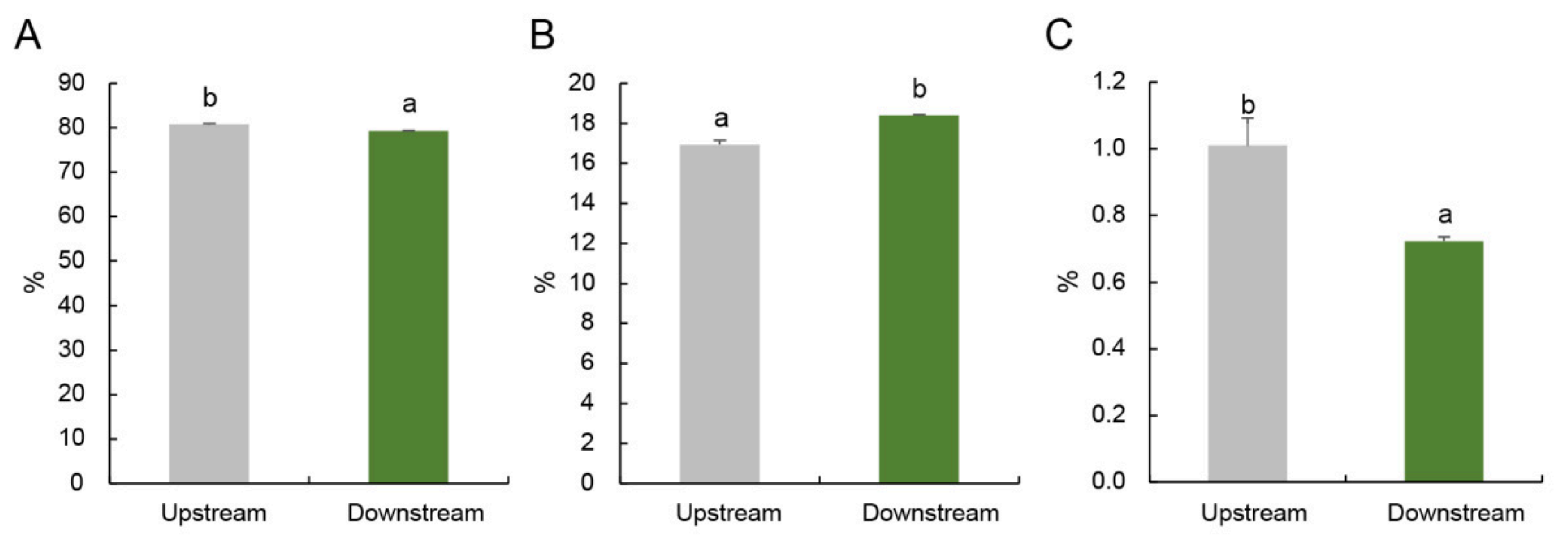
Comparison of muscle nutritional composition between upstream and downstream *H. guttatus*. (**A**) Moisture content; (**B**) crude protein content; and (**C**) crude lipid content. Different lowercase letters indicate significant differences between groups (*p* < 0.05).

Nutritional composition analysis of dorsal muscle tissues revealed significant differences between upstream and downstream populations of *H. guttatus* (Figure 13). Upstream individuals showed significantly higher moisture and crude lipid contents compared with downstream fish, whereas downstream individuals exhibited significantly higher crude protein content. These results indicate habitat-associated variation in muscle nutritional composition, suggesting differences in energy storage and nutrient allocation patterns between fish populations inhabiting distinct river sections.

## 4. Discussion

### 4.1. Spatial Divergence in Dietary Composition Between Upstream and Downstream Habitats

The present study demonstrated clear spatial differences in the dietary composition of *H. guttatus* inhabiting upstream and downstream habitats of the Datengxia Water Conservancy Project. Both 12S and COI metabarcoding analyses revealed significant divergence in prey utilization patterns, indicating that fish from the two habitats relied on distinct trophic resources. Interestingly, the two molecular markers revealed different patterns of dietary diversity. The 12S dataset showed significantly higher prey diversity in upstream fish, whereas COI metabarcoding indicated higher diversity of detected metazoan prey in downstream individuals. These contrasting patterns should not necessarily be interpreted as contradictory, because 12S and COI differ in primer affinity, taxonomic coverage, amplification efficiency, amplicon characteristics, and reference-database representation. The MiFish 12S marker is particularly effective for detecting fish-derived DNA and therefore provides relatively high resolution for vertebrate prey, whereas the COI marker offers broader coverage of metazoan taxa and is more effective for detecting many invertebrate groups, including crustaceans, annelids, and aquatic insects [31,32,33]. Consequently, the two markers characterize partially different components of the diet.

Importantly, sequence proportions generated by 12S and COI should not be directly pooled or interpreted as quantitatively equivalent estimates of prey biomass because marker-specific amplification biases can affect the relative representation of different prey taxa. Instead, the two datasets should be interpreted as complementary sources of dietary information. In the present study, the 12S results primarily revealed differences in fish-prey composition, whereas the COI dataset broadened the detectable prey spectrum by capturing a greater diversity of non-fish metazoans. Taken together, these findings suggest that upstream fish utilized a more diverse assemblage of fish prey, while downstream fish showed greater diversity among the broader metazoan prey detected by COI. Thus, the apparent inconsistency between the two markers likely reflects their complementary taxonomic detection ranges rather than conflicting ecological signals.

The very high PERMANOVA R^2^ values observed for both dietary metabarcoding datasets should nevertheless be interpreted with caution. PERMDISP analyses detected significant differences in multivariate dispersion between upstream and downstream groups for both 12S and COI, particularly for the COI dataset. This indicates that the significant PERMANOVA results may reflect not only differences in the location of group centroids but also differences in within-group variability. Notably, downstream samples showed greater dispersion for both dietary markers. Therefore, although the combined ordination, taxonomic composition, and differential-abundance analyses consistently demonstrate pronounced spatial structuring of dietary profiles, the magnitude of the upstream–downstream effect inferred from PERMANOVA alone should not be overinterpreted.

Taxonomic composition analyses further supported the spatial differentiation observed between upstream and downstream fish. Upstream samples were enriched with taxa such as *Sinibotia robusta* and several benthic-associated groups, whereas downstream samples contained higher abundances of *Cyprinus carpio*, *Megalobrama terminalis*, *Macrobrachium nipponense*, and arthropod-related taxa. These patterns demonstrate differences in prey use between fish collected from the two river sections. The contrasting hydrodynamic settings of the two river sections provide a plausible physical context for these dietary differences. The upstream reach represents an impounded environment with reduced lotic characteristics and relatively longer water residence, whereas the downstream reach is more directly influenced by regulated discharge and associated flow fluctuations. Such differences in flow regime may affect sediment transport, retention and redistribution of particulate organic matter, and the spatial availability of benthic and drifting prey, thereby potentially altering prey accessibility to fish. However, because ambient prey availability, hydrological conditions, water-quality variables, and sediment characteristics were not quantified, the mechanisms underlying these dietary differences cannot be determined from the present data. The observed patterns should therefore be interpreted as spatial associations with sampling location rather than as direct effects of dam regulation or habitat-driven changes in prey availability.

From an aquaculture perspective, the habitat-associated dietary differences identified in wild *H. guttatus* populations may provide useful references for nutritional management under artificial culture. The habitat-associated dietary differences identified in wild *H. guttatus* populations may provide baseline ecological information for future nutritional studies under artificial culture. However, the present metabarcoding data do not directly determine optimal feed ingredients or nutrient requirements. Controlled feeding experiments will therefore be necessary before these dietary patterns can be translated into practical feed formulation strategies.

### 4.2. Gut Microbial Restructuring and Functional Differentiation Associated with Habitat Variation

The gut microbiota of *H. guttatus* also differed markedly between upstream and downstream habitats. Upstream fish exhibited significantly higher microbial richness, diversity, and phylogenetic diversity, suggesting the presence of a more complex intestinal microbial ecosystem. Although upstream and downstream samples occupied distinct positions in the ordination space, the PERMANOVA test did not reach statistical significance (R^2^ = 0.894, *p* = 0.100). PERMDISP was likewise non-significant (F = 69.99, *p* = 0.100). Therefore, the beta-diversity pattern should be interpreted as an apparent spatial differentiation trend rather than statistically confirmed separation in overall microbial community structure. At the taxonomic level, upstream fish were characterized by increased abundance of Firmicutes-related taxa, including Lactococcus, Clostridium, and Paraclostridium, whereas downstream fish showed enrichment of Proteobacteria-related taxa such as Plesiomonas and *Plesiomonas shigelloides*. The higher abundance of Firmicutes-related taxa in upstream fish may indicate enhanced microbial functions associated with organic matter utilization and nutrient metabolism. In contrast, the enrichment of Proteobacteria-related taxa in downstream individuals may be associated with differences between the two sampling environments and their corresponding resource-use patterns [34,35,36,37]. Hydrodynamic differences may also indirectly contribute to the distinct microbial patterns observed between the two river sections. Flow regulation can influence the transport and distribution of suspended particles, environmental microorganisms, and food resources, while also modifying feeding opportunities and prey composition. These processes may alter both the microbial exposure experienced by fish and the substrates entering the intestine through feeding, thereby providing a potential pathway linking hydrodynamic conditions with gut microbial variation. However, because environmental conditions and food-resource availability were not directly quantified, the factors responsible for these microbial differences remain unresolved. The enrichment of Firmicutes-related taxa in upstream fish, together with the increased abundance of Plesiomonas in downstream individuals, therefore indicates distinct gut microbial configurations associated with the two sampling locations, rather than demonstrating a direct effect of habitat conditions or dam regulation.

Functional prediction analyses further supported the observed compositional differences. PICRUSt2 indicated that downstream microbial communities were enriched in pathways associated with membrane transport, xenobiotics biodegradation, lipid metabolism, and environmental adaptation, which may be consistent with differences in environmental exposure between the two river sections [38,39]. In contrast, upstream microbiota showed relatively higher abundance of pathways related to replication and repair, nucleotide metabolism, and cofactor metabolism, indicating comparatively stable metabolic functions. Similarly, FAPROTAX prediction showed that upstream microbial communities were associated with fermentation, nitrogen cycling, sulfur metabolism, and chemoheterotrophy, whereas downstream communities were enriched in functions related to hydrocarbon degradation, aromatic compound degradation, and human pathogen-associated pathways. BugBase phenotype prediction also revealed significantly higher abundances of stress-tolerant, biofilm-forming, anaerobic, and potentially pathogenic phenotypes in downstream fish. Collectively, these functional differences indicate that habitat-associated microbial communities differ not only in taxonomic composition but also in their predicted metabolic potential. Such variation may provide useful references for future nutritional management of *H. guttatus*, as microbial functions involved in nutrient metabolism and intestinal homeostasis are increasingly recognized as important components of fish health and feeding performance. thereby providing ecological references for microbiota-informed feed development. However, experimental studies are still required to determine whether these naturally occurring microbial differences can be translated into optimized dietary strategies under aquaculture conditions.

Network analysis further demonstrated that habitat-associated microbial restructuring involved not only changes in bacterial abundance but also alterations in potential ecological interactions among dominant taxa [40,41]. Core genera such as Cetobacterium, Lactococcus, Clostridium, and Plesiomonas occupied central positions within the network, suggesting important roles in maintaining intestinal microbial stability. From an aquaculture perspective, these microbial signatures may serve as useful indicators for nutritional management. Taxa enriched in upstream fish, including Lactococcus and several Firmicutes-related genera, may represent candidates for future functional investigation. However, their potential probiotic properties cannot be inferred from relative abundance alone and would require isolation, functional characterization, safety assessment, and controlled feeding trials. Although these implications require experimental validation, the present results provide a microbial baseline for developing habitat-informed feeding strategies and microbiota-based nutritional management of *H. guttatus*.

### 4.3. Nutritional Implications and Study Limitations

In addition to dietary and microbial differences, muscle nutritional composition also varied between upstream and downstream populations. Upstream fish contained higher crude lipid levels, whereas downstream individuals exhibited greater crude protein content, suggesting different patterns of nutrient allocation under contrasting habitat conditions. These differences coincided with spatial variation in dietary composition and intestinal microbial communities; however, the present observational design does not allow the underlying causes of muscle nutritional differences to be determined [42,43]. Hydrodynamic conditions may also contribute indirectly to these nutritional differences by influencing prey accessibility, feeding opportunities, and the energetic costs associated with movement and habitat use. However, because individual energy expenditure and hydrodynamic exposure were not measured, these potential relationships remain hypothetical. Natural dietary composition, gut microbiota, and muscle nutritional characteristics are closely interconnected, collectively reflecting the nutritional status of wild fish populations [36]. Therefore, the integrated approach employed in this study provides not only ecological insights into habitat-associated variation, but also valuable information for aquaculture nutrition. Specifically, the distinct dietary profiles identified in different habitats may provide references for future feed formulation, while differences in gut microbial communities provide a baseline for future functional studies of host-associated microbiota and intestinal health [44]. Moreover, the observed variation in muscle nutritional composition suggests that population-specific pre-conditioning diets prior to broodstock domestication, captive breeding, or stock enhancement may benefit from considering the habitat origin of founder populations. Although these practical implications require validation through controlled feeding experiments, the present findings establish an ecological foundation for developing habitat-informed nutritional strategies for *H. guttatus* aquaculture.

Several limitations should nevertheless be acknowledged. First, the sample size was relatively limited, which may reduce statistical power and increase uncertainty in some community-level analyses. Because the wild population of *H. guttatus* is nationally protected in China, field sampling followed a conservative strategy to minimize disturbance to the natural population, which constrained the number of individuals that could be collected. Future studies should increase biological replication where permitted and incorporate repeated temporal sampling to assess the robustness of the observed spatial patterns. In addition, individual biometric and demographic characteristics, including body length, body weight, sex, and age, were not systematically recorded during field sampling. Therefore, potential effects of individual biological variation on dietary composition, gut microbiota, and muscle nutritional traits cannot be completely excluded. Therefore, potential ontogenetic and reproductive variation among individuals may have contributed to the observed differences in dietary composition, gut microbiota, and muscle nutritional traits and cannot be completely excluded as confounding factors. Furthermore, because all samples were collected during a single sampling period in September, the present results represent upstream–downstream spatial patterns during this sampling period, and potential seasonal variation cannot be evaluated from the current dataset. Future studies should incorporate standardized measurements of body size, sex, and age and, where appropriate, include these variables as covariates in statistical analyses. Moreover, because the dietary, gut microbiota, and muscle nutritional datasets could not be reliably matched at the individual level, direct correlation analyses among these three components were not feasible. Future studies should adopt individual-level sample tracking to enable integrated analyses of diet–microbiota–nutrition relationships. Second, environmental variables such as nutrient concentrations, dissolved oxygen, sediment characteristics, hydrological parameters and ambient prey-resource availability were not measured, preventing direct assessment of the environmental and trophic drivers underlying the observed biological differences. Consequently, the differences identified in this study should be interpreted as spatial associations between upstream and downstream populations rather than as direct causal effects of dam regulation. Third, metabarcoding-based dietary analyses may be influenced by primer bias, prey DNA degradation, and database limitations, particularly at the species level [45]. Finally, PICRUSt2, FAPROTAX, and BugBase analyses provide predictive rather than experimentally validated functions, and therefore the functional interpretations should be considered preliminary. From an applied perspective, the ecological differences identified in wild *H. guttatus* populations should not be regarded solely as habitat-specific ecological phenomena, but also as valuable biological references for developing future nutritional strategies in aquaculture and conservation breeding. Future studies combining individual-level sample tracking, environmental monitoring, metagenomics, metabolomics, and controlled feeding experiments would help clarify the mechanistic relationships among habitat variation, dietary shifts, gut microbial dynamics, and muscle nutritional characteristics in freshwater fishes.

## 5. Conclusions

This study revealed clear upstream–downstream variation in dietary composition, gut microbial characteristics, and muscle nutritional traits of *H. guttatus* inhabiting the Datengxia dam-regulated river system. The two populations differed in prey-use patterns, intestinal microbial composition, predicted microbial functions, and muscle nutritional allocation, with upstream fish generally showing higher microbial diversity and crude lipid content, whereas downstream fish exhibited distinct dietary profiles, greater representation of Proteobacteria-associated taxa, and higher crude protein content. These observed differences should be interpreted as spatial associations between upstream and downstream populations within a dam-regulated river system rather than as direct causal effects of dam regulation. These findings demonstrate that *H. guttatus* populations from upstream and downstream habitats represent biologically distinct source groups within the same regulated river system. Therefore, habitat origin should be considered when selecting wild individuals for artificial propagation, broodstock development, stock enhancement, or conservation programs. More broadly, the results highlight the importance of evaluating upstream and downstream populations separately when assessing the ecological and nutritional characteristics of fish inhabiting dam-regulated rivers.

## Data Availability

The raw sequencing data generated in this study have been deposited in the NCBI Sequence Read Archive (SRA) under BioProject accession number PRJNA1481365.

## Supplementary Materials

The following supporting information can be downloaded at https://www.mdpi.com/article/10.3390/ani16182818/s1: Figure S1: Functional annotation of the intestinal bacterial communities based on the FAPROTAX database. Differentially enriched ecological functions between upstream and downstream *H. guttatus* are presented. Bars represent the mean relative abundance of each functional category, and dots indicate the differences between groups with 95% confidence intervals; Figure S2: Comparison of alpha diversity indices for prey communities identified by 12S and COI metabarcoding in upstream and downstream *H. guttatus*. ACE and Simpson indices are shown for each marker. Statistical differences between upstream and downstream groups were evaluated using Student’s *t*-test, Welch’s *t*-test, or the Mann–Whitney U test, as appropriate according to the assumptions of normality and homogeneity of variance. *p* values for comparisons between the upstream and downstream groups are shown directly in each panel, with *p* < 0.05 considered statistically significant; Figure S3: Functional annotation of the intestinal bacterial communities based on the FAPROTAX database. Differentially enriched ecological functions between upstream and downstream *H. guttatus* are presented. Bars represent the mean relative abundance of each functional category, and dots indicate the differences between groups with 95% confidence intervals. *p* values for comparisons between the upstream and downstream groups are shown directly in each panel, with *p* < 0.05 considered statistically significant; Table S1: Summary statistics of sequencing data generated from 12S, COI, and 16S rRNA gene datasets.
