# Supplementary material for: Habitat-Associated Variation in Dietary Composition, Gut Microbiota, and Muscle Nutritional Characteristics of Hemibagrus guttatus in a Dam-Regulated River
Source: Animals (Basel). 2026 Sep 8;16(18):2818. doi: 10.3390/ani16182818 (PMC13603508; doi:10.3390/ani16182818)
Supplement: Supplementary file 1 [file animals-16-02818-s001.zip › Figure S1-S3.pdf]

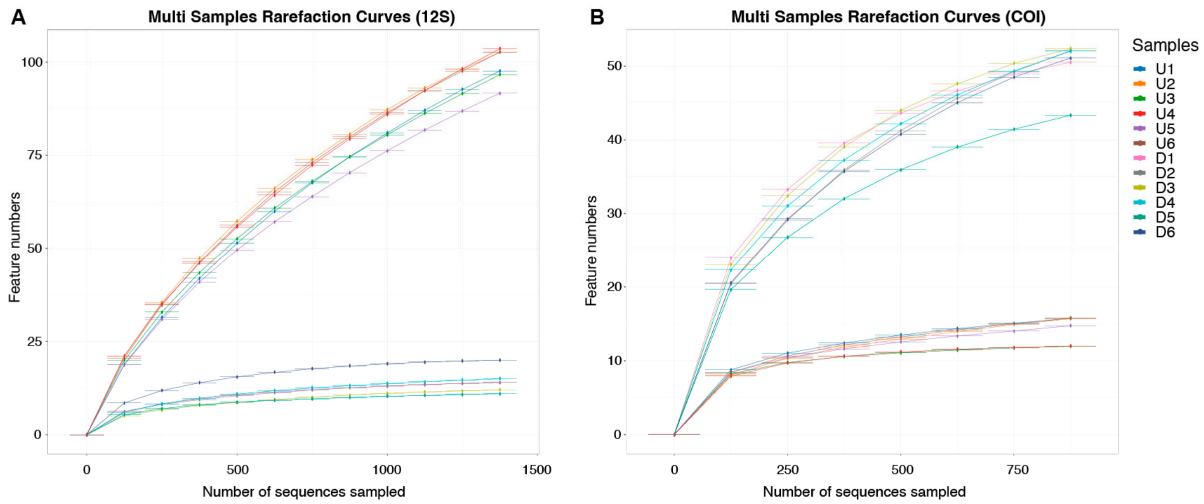

Figure S1. Functional annotation of the intestinal bacterial communities based on the FAPROTAX database. Differentially enriched ecological functions between upstream and downstream *H. guttatus* are presented. Bars represent the mean relative abundance of each functional category, and dots indicate the differences between groups with 95% confidence intervals.

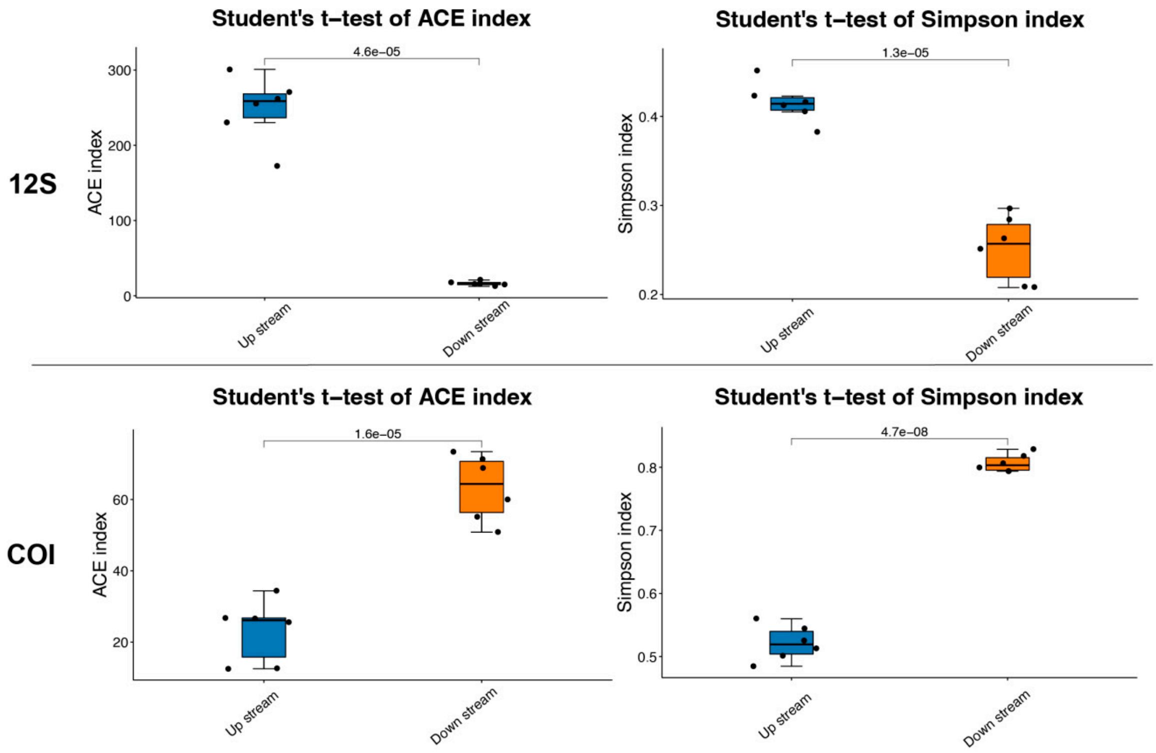

Figure S2. Comparison of alpha diversity indices for prey communities identified by 12S and COI metabarcoding in upstream and downstream *H. guttatus*. ACE and Simpson indices are shown for each marker. Statistical differences between upstream and downstream groups were evaluated using Student's t-test, Welch's t-test, or the Mann–Whitney U test, as appropriate according to the assumptions of normality and homogeneity of variance. *P* values for

comparisons between the upstream and downstream groups are shown directly in each panel, with $P < 0.05$ considered statistically significant.

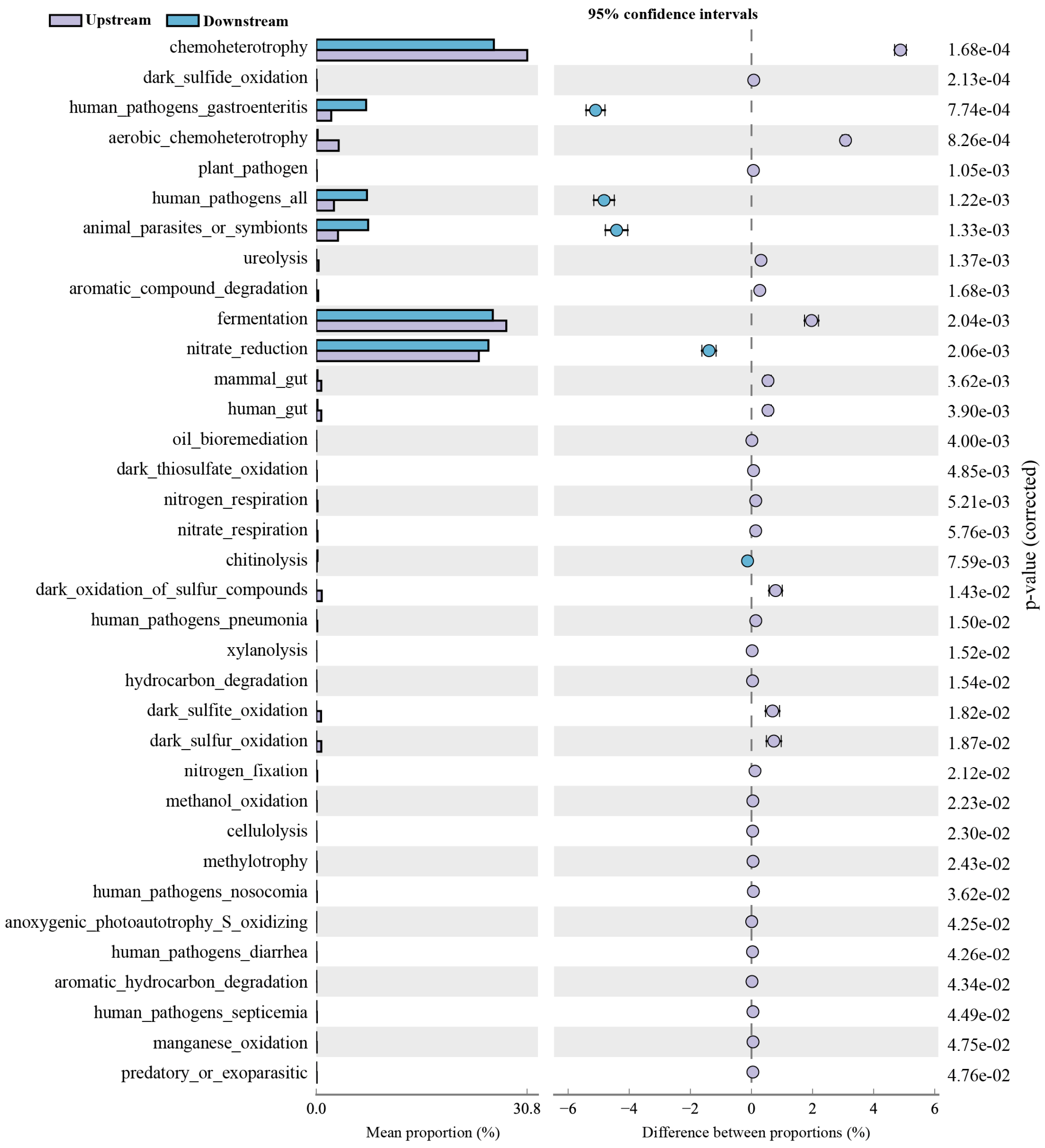

Figure S3. Functional annotation of the intestinal bacterial communities based on the FAPROTAX database. Differentially enriched ecological functions between upstream and downstream *H. guttatus* are presented. Bars represent the mean relative abundance of each functional category, and dots indicate the differences between groups with 95% confidence intervals. *P* values for comparisons between the upstream and downstream groups are shown directly in each panel, with $P < 0.05$ considered statistically significant.
